# Alleviation of Depression by Glucagon-Like Peptide 1 Through the Regulation of Neuroinflammation, Neurotransmitters, Neurogenesis, and Synaptic Function

**DOI:** 10.3389/fphar.2020.01270

**Published:** 2020-08-14

**Authors:** Young-Kook Kim, Oh Yoen Kim, Juhyun Song

**Affiliations:** ^1^Department of Biochemistry, Chonnam National University Medical School, Hwasun, South Korea; ^2^Department of Food Science and Nutrition, Dong-A University, Busan, South Korea; ^3^Center for Silver-targeted Biomaterials, Brain Busan 21 Plus Program, Graduate School, Dong-A University, Busan, South Korea; ^4^Department of Anatomy, Chonnam National University Medical School, Hwasun, South Korea

**Keywords:** depression, glucagon-like peptide-1 (GLP-1), neuroinflammation, neurogenesis, synaptic plasticity

## Abstract

Depression has emerged as a major cause of mortality globally. Many studies have reported risk factors and mechanisms associated with depression, but it is as yet unclear how these findings can be applied to the treatment and prevention of this disorder. The onset and recurrence of depression have been linked to diverse metabolic factors, including hyperglycemia, dyslipidemia, and insulin resistance. Recent studies have suggested that depression is accompanied by memory loss as well as depressive mood. Thus, many researchers have highlighted the relationship between depressive behavior and metabolic alterations from various perspectives. Glucagon-like peptide-1 (GLP-1), which is secreted from gut cells and hindbrain areas, has been studied in metabolic diseases such as obesity and diabetes, and was shown to control glucose metabolism and insulin resistance. Recently, GLP-1 was highlighted as a regulator of diverse pathways, but its potential as the therapeutic target of depressive disorder was not described comprehensively. Therefore, in this review, we focused on the potential of GLP-1 modulation in depression.

## Introduction

Depression is emerging as one of the main causes of morbidity and mortality globally, and the number of individuals reported to be afflicted with the disorder continues to increase (Ledford, 2014; Gerhard et al., 2016). Even though extensive research has been conducted, depression remains incompletely understood owing to the scarcity of biomarkers and the heterogeneity of precipitating causes, such as chronic stress (Kessler et al., 2005; Krishnan and Nestler, 2008). A recent study investigated the relationship between depression and metabolism, and found that metabolites, such as advanced glycation end products, in patients with depression are altered compared to those in controls (Setoyama et al., 2016; Akimoto et al., 2019).

Glucagon-like peptide-1 (GLP-1) is a peptide hormone primarily synthesized in the gut that circulates through the blood, and plays a critical role in the regulation of glucose metabolism in the gut-brain axis. GLP-1 is also secreted by a small population of neurons in the hindbrain (Richard et al., 2015). GLP-1 receptors (GLP-1Rs) are distributed across various organs including the pancreas, lung, stomach, intestine, kidney, heart, and diverse brain regions (Bullock et al., 1996; Li et al., 2009; Campbell and Drucker, 2013; Heppner et al., 2015a).

**Figure 1:**
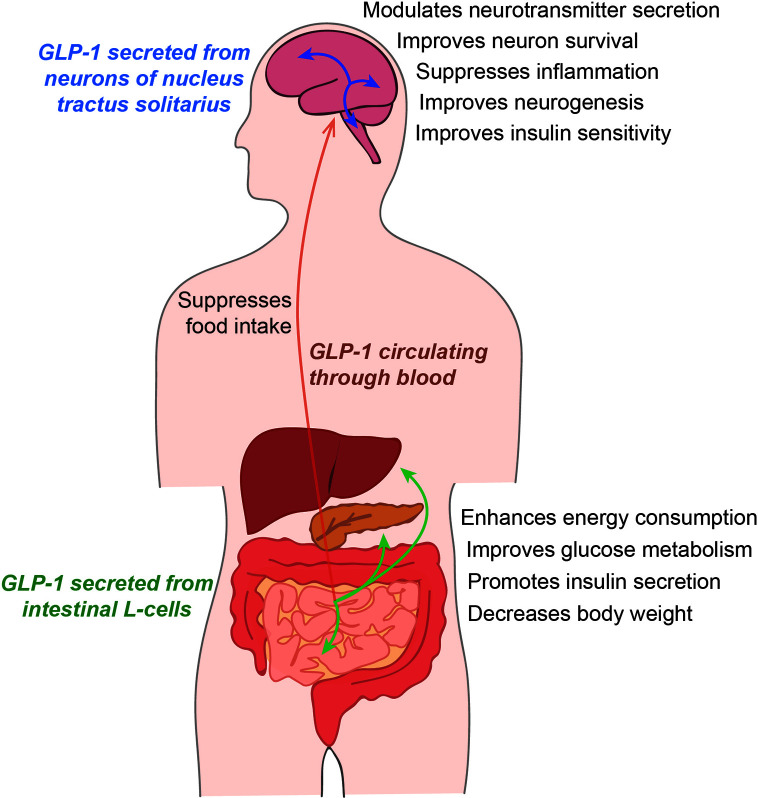
The function of glucagon-like peptide 1 (GLP-1) in humans. GLP-1, secreted from the intestinal L-cells, circulates the whole body through the blood. GLP-1 influences energy metabolism and glucose metabolism by regulating the insulin level. In the brain, GLP-1 secreted from intestinal cells can be absorbed into the brain, and the GLP-1 secreted from neurons remains in the cerebrospinal fluid. GLP-1 can control the secretion of various neurotransmitters and the progression of neuroinflammation, and can regulate insulin sensitivity in the brain.

In the central nervous system (CNS), GLP-1 and GLP-1R agonists are diffused into the cerebrospinal fluid (CSF) and the brain as demonstrated in rodent models (Wang et al., 2015), and increased GLP-1 concentration in the brain is thought to influence brain function in the hippocampus (Kastin et al., 2002; Kastin and Akerstrom, 2003; McClean et al., 2010; Hunter and Holscher, 2012). Glucose-insulin metabolic homeostasis is important for the gut-brain axis because glucose is used as the main source of energy in the brain (Weber, 2016). Major incretins such as GLP-1 and GIP can control appetite by reducing feelings of hunger and by increasing satiety and have a regulatory effect on glucose homeostasis through the reduction of blood glucose levels and insulin release (**Figure 1**) (Edholm et al., 2010; Duarte et al., 2013).

Although previous studies have demonstrated that GLP-1 has positive effects on attenuating neuropathology, its function and specific actions in CNS diseases have not been reviewed comprehensively. Here, we review the therapeutic effects of GLP-1 on depression from a variety of perspectives and highlight the promising, beneficial roles of GLP-1 as the therapeutic regulator of impaired neurogenesis, neuroinflammation, imbalance of neurotransmitter secretion, and synaptic dysfunction in the depressive brain.

## What Is GLP-1?

GLP-1 is a 30 amino acid-long peptide hormone mainly produced in the intestinal L-cells of the gut that is secreted into the blood (**Figure 1**) (Habib et al., 2013; Richard et al., 2015). GLP-1 is also secreted from microglia (Kappe et al., 2012) and specific neurons of the nucleus tractus solitarius (NTS) (Alhadeff et al., 2012). Activation of these GLP-1-secreting neurons is regulated by the glucose level. The neurons innervate several brain regions including the hypothalamus, thalamus, paraventricular nucleus, cortex, and arcuate nucleus (Cork et al., 2015), and convey vagal motor information to the pancreas (Parker et al., 2010; Kappe et al., 2013; Calsolaro and Edison, 2015; Richard et al., 2015). GLP-1 has been reported to improve glucose-dependent insulin action through the G-protein-coupled receptor, GLP-1R (Drucker and Nauck, 2006).

GLP-1 is involved in the regulation of energy balance (Holst, 2007), and its activation and subsequent binding to the GLP-1R reduces food intake and, consequently, body weight (Turton et al., 1996). In the CNS, GLP-1Rs are expressed by neurons in the hippocampus, which is known to be a cognition-related brain region, and have been observed in neocortical pyramidal neurons (Hamilton and Holscher, 2009). In the brain, GLP-1 secreted from intestinal cells can be absorbed into the brain, and GLP-1 secreted from neurons remains in the CSF (Cabou and Burcelin, 2011). GLP-1 can control the secretion of various neurotransmitters, the progression of neuroinflammation, and the increase in insulin sensitivity in the brain (Athauda and Foltynie, 2016). GLP-1 is also thought to play an important role in glucose metabolism and neuronal function, including synaptic plasticity and neuronal metabolism, in the brain (Holscher, 2012). One study demonstrated that overexpression of GLP-1R in the hippocampus leads to an improvement in memory function (McClean and Holscher, 2014; Hansen et al., 2015). Microglia are also known to secrete GLP-1(Kappe et al., 2012). Furthermore, the activity of GLP-1 secreting neurons is regulated by the glucose level (Parker et al., 2010; Kappe et al., 2013).

A recent study demonstrated that GLP-1 has a neurotrophic effect and promotes neurotrophic processes in the brain (Mainardi et al., 2015). Robinson et al. demonstrated that a mixture of GLP-1 agonists boosts memory and improves insulin action in the brain (Robinson et al., 2019). Exendin-4, a stable synthetic form of GLP-1, is commonly used clinically for diabetes treatment because GLP-1 has a short half-life (Drucker and Nauck, 2006; Heppner et al., 2015b). Furthermore, exendin-4 can cross the blood-brain barrier (Kastin and Akerstrom, 2003) and has a neuroprotective role in some neurological disorders (Holscher, 2010; Candeias et al., 2015). Clinically, GLP-1 analogs have been developed to treat diabetes and obesity, given that they have beneficial effects on blood glucose control and on the cardiovascular system. Liraglutide is a GLP-1 analog used in clinically obese patients for reducing body weight (Crane and McGowan, 2016). The effects of weight loss were observed upon daily liraglutide injection (Mora and Johnson, 2017), oral administration of liraglutide (3 mg) (Manigault and Thurston, 2016), and subcutaneous treatment of liraglutide for 20 weeks (1.2 to 3 mg) (Astrup et al., 2009). An interesting study also showed that oral administration of GLP-1 could reduce depression risk caused by hyperglycemia in a patient with type 2 diabetes (Onoviran et al., 2019).

In the next part of this review, the diverse roles of GLP-1 in the depressive brain will be discussed. Moreover, in each section, we will describe the various anti-depressive effects of GLP-1 in the CNS based on recent evidence.

## Overview of Depression

Major depressive disorder (MDD) is a highly prevalent mood disorder characterized by ruminative thoughts, impaired cognition and anhedonia, and attentional control deficits (Marchetti et al., 2012; Disease et al., 2016). Depression is thought to arise from a combination of genetic background and environmental stressors (Belmaker and Agam, 2008). Depression is considered a severe disorder because the features of depression negatively influence the quality of life of patients and their families, and are associated with increased economic and mental health burden (Hamilton et al., 2015).

Some reports have demonstrated that more than 50% of patients with depression experience chronic and recurrent problems throughout their lifetime, and that depressive symptoms can gradually aggravate and lead to suicide if patients are not treated with proper drugs or counseling (Monroe and Harkness, 2005; Rush et al., 2006). For these reasons, patients with depression must be accurately treated, and research aimed at preventing the onset of depression is therefore critical.

Depression is a disease that is highly related to neuroinflammation, neurotransmitter imbalance, blood-brain barrier hyperpermeability, deficits in neurogenesis, and synaptic dysfunction (Najjar et al., 2013; Felger and Treadway, 2017). One study observed that patients with depression showed impaired neurogenesis, neural growth retardation, and synaptic plasticity reduction (Serafini, 2012). In particular, the prefrontal cortex (PFC), amygdala, and hippocampus are crucial for regulating emotion, stress responses, and motivation. In patients with depression, the function of the PFC and hippocampus is disrupted, whereas the amygdala is hyperactivated (Serafini, 2012).

Although numerous anti-depressive treatments are available, 30% of patients diagnosed with depression do not experience positive therapeutic effects with these therapies (Al-Harbi, 2012). Hence, the mechanism of depression must be elucidated to facilitate the discovery of novel and effective anti-depressive therapies.

The gut is a key member of the gut-brain axis owing to its own enteric nervous system and its independent responses to the exterior environment and stress (Rao and Gershon, 2016). In recent years, the gut-brain axis has been increasingly considered a promising topic for the study of CNS-related diseases because the gut microbiota and gut hormones have been shown to interact with other organs including the brain (Knight et al., 2017), although the mechanisms involved in gut homeostasis and brain function are as yet uncharacterized (Collins and Bercik, 2009; Neufeld and Foster, 2009). Previous studies have demonstrated that the regulation of the immune system, endocrine system, and nervous system is strongly involved in the connection between the gut and the brain (Forsythe et al., 2010).

Several studies have shown that depressed patients have impaired gut-brain axis metabolism, appetite disturbances, and gut hormone abnormality (Collins and Bercik, 2009; Evrensel and Ceylan, 2015; Jiang et al., 2015). For these reasons, the current view of depression as an exclusively neurological disorder was expanded to consider it a systemic disease, given that depression involves the impairment of the hypothalamic−pituitary−adrenal (HPA) axis, immune dysregulation, and disturbance of the gut-brain axis (Maes et al., 2011; O’Mahony et al., 2015). Various factors and signaling pathways are linked to gut-brain axis dysfunction that putatively leads to depression (Scott et al., 2013).

Although there is evidence associating depression with the gut, the action of the gut hormone GLP-1 is still not fully understood in the depressive brain. In the next section, we will focus on the diverse roles of GLP-1 in depression (**Table 1**).

**Table 1:** Possible association between glucagon-like peptide 1 (GLP-1) and depression based on previous studies.

Experiment target	Result	Reference
GLP-1 or exendin-4	Influenced the dopamine system and reduced food reward behavior	(Richard et al., 2015)
Liraglutide	Exerted antipsychotic effect	(Dixit et al., 2013)
Exendin-4	Reduced cocaine self-administration	(Sorensen et al., 2015)
Exendin-4	Elevated turnover of dopamine through dopamine receptor 2 signaling	(Anderberg et al., 2014)
GLP-1R activation	Stimulated the release of GABA, glutamate, serotonin, and dopamine	(Rebosio et al., 2018)
Liraglutide or exendin-4	Enhanced neurogenesis and neural proliferation	(Bertilsson et al., 2008; Li et al., 2009; Isacson et al., 2011; Darsalia et al., 2012; McGovern et al., 2012; Porter et al., 2013; Parthsarathy and Holscher, 2013a; Holscher, 2014; Darsalia et al., 2014b; Sango and Utsunomiya, 2015)
(Val(8))GLP-1-Glu-PAL	Increased hippocampal neurogenesis and cell proliferation	(Lennox et al., 2013)
P7C3 (aminopropyl carbazole compound)	Activation of cAMP/PKA and Akt/GSK3 signaling	(Wang et al., 2018)
Liraglutide	Promoted neurite outgrowth through Wnt signaling	(He et al., 2018)
Liraglutide	Enhanced synaptic plasticity and attenuated depressive behavior	(Weina et al., 2018)
Liraglutide	Improved cognitive function and depressive symptoms in patients	(Cuomo et al., 2018)
Sitagliptin	Enhanced cognitive function and protected neurons against oxidative stress	(Gault et al., 2015)
Liraglutide	Activated LTP and improved cognitive dysfunction	(McClean et al., 2011)
GLP-1R knockout mouse	Impaired synaptic plasticity and memory formation	(Abbas et al., 2009)
GLP-1R overexpression mouse	Enhanced learning and neuroprotection	(During et al., 2003)
GLP-1 or exendin-4	Chronic administration reduced depression-like behavior	(Anderberg et al., 2016)

### Neuroinflammation in Depression and Its Relation to GLP-1

**Figure 2:**
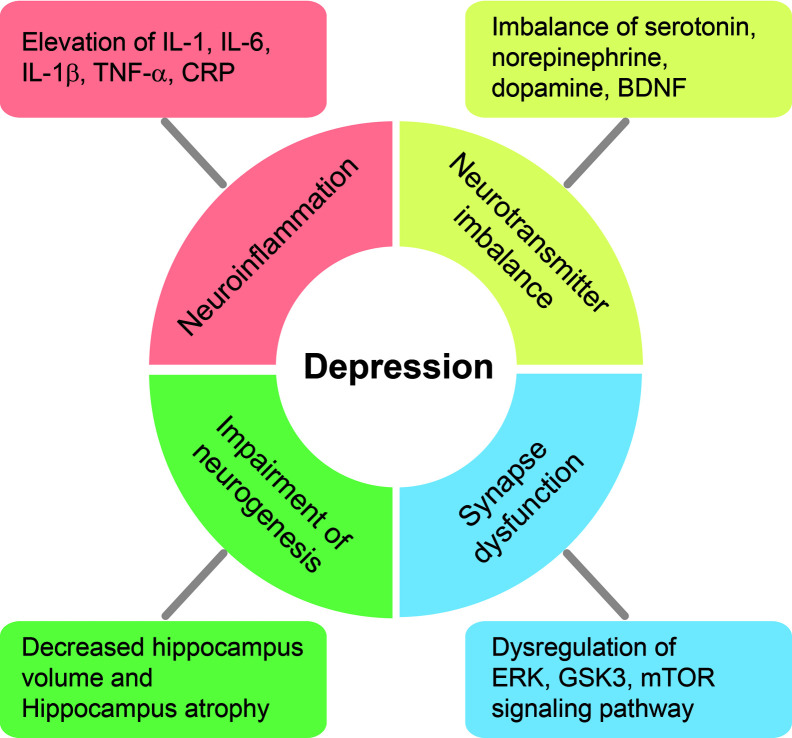
Schematic diagram of physiological and molecular changes in the brain of patients with depression. We have summarized several physiological and molecular alterations in the brain of patients with depression. The depressive brain exhibits severe neuroinflammation involving higher pro-inflammatory cytokine production, neurotransmitter imbalance, and synaptic dysfunction through the dysregulation of ERK, Glycogen synthase kinase 3β (GSK-3β), and mammalian target of the rapamycin (mTOR) pathways, and these ultimately lead to the loss of hippocampal volume and cognitive decline.

The immune system in the CNS has been shown to regulate brain development, neurogenesis, mood, and behavior (do Prado et al., 2016; Zheng et al., 2016; de Miranda et al., 2017). Over the past two decades, several studies have indicated that neuroinflammation is a critical reason for the onset, deterioration, relapse, and maintenance of depression (**Figure 2**) (Kopschina Feltes et al., 2017; Paudel et al., 2018; Woelfer et al., 2019). A recent study demonstrated that neuroinflammation could evoke depressive symptoms such as anhedonia and motor retardation (Felger and Treadway, 2017). Chronic neuroinflammation has been reported to contribute to serotonergic, dopaminergic, and noradrenergic dysfunction in the brain (Song and Wang, 2011) and to lead to chronic immune dysregulation (Carvalho et al., 2013).

Previous studies found that pro-inflammatory cytokines, including tumor necrosis factor-α (TNF-α) and interleukin (IL)-6, IL-1β, and C-reactive protein (CRP), are markedly increased in the blood and CSF of patients with depression (Hestad et al., 2016; Smith et al., 2018). IL-1, IL-6, IL-1β, TNF-α, and CRP are considered markers of the initiation, relapse, and progression of depression (**Figure 2**) (Haapakoski et al., 2016). Clinically, elevated levels of CRP and IL-6 were observed in patients with depression when compared to healthy subjects (Goldsmith et al., 2016) and were associated with cognitive symptoms in such patients (Gimeno et al., 2009). Furthermore, these increased levels of pro-inflammatory cytokines resulted in an increase of corticotropin-releasing hormone activity and hyperactivity of the HPA axis in most patients with depression (Kopschina Feltes et al., 2017). Excessive pro-inflammatory cytokines suppress the negative feedback of the HPA axis, boost the permeability of the blood-brain barrier, decrease the synthesis of neurotransmitters such as serotonin (5-HT), and ultimately lead to the onset of depression (Haroon and Miller, 2017; Leonard, 2018). Moreover, higher concentrations of several chemokines, such as CCL2 and CXCL8, were detected in the serum and CSF of depressed patients (Sutcigil et al., 2007; Piletz et al., 2009; Black and Miller, 2015; Khandaker et al., 2015; Slusarczyk et al., 2016). Previous studies have demonstrated that the proliferation of mononuclear cells is suppressed and T cell mitogens in the blood are decreased in patients with severe depression (Nunes et al., 2002; Irwin and Miller, 2007), and that impaired cell-mediated immunity is involved in the pathophysiology of depression (Kohler et al., 2017). Other studies have demonstrated a strong correlation between Th_17_ immune cells and the progression of depression (Korn et al., 2009; Slyepchenko et al., 2016).

As mentioned above, inflammatory responses and immunity are involved in the development and onset of depression (**Figure 2**). Modulation of inflammation may thus mitigate depressive behavior in patients with depression.

GLP-1 has been reported to promote the production of anti-inflammatory cytokines in various organs including the adipose tissue and pancreas, and the brain (Dobrian et al., 2011; Lee et al., 2012; Darsalia et al., 2014a; Augestad et al., 2020; Reed et al., 2020). In addition, GLP-1 can boost immune cell infiltration as well as the production of pro-inflammatory cytokines under inflammatory conditions (Liu et al., 2009; Parthsarathy and Holscher, 2013b). One study demonstrated that exendin-4 (50 nM) treatment reduces the expression of pro-inflammatory genes such as NF-κB p65 and the TNF receptor superfamily member 1A in human pancreatic islet cells (Velmurugan et al., 2012). In addition, treatment of a DPP-4 inhibitor in diabetic mice increased the levels of anti-inflammatory cytokines, and increased the activation of regulatory T cells, indicating an involvement in the pathology of diabetes (Tian et al., 2010). Another study has reported that treatment of the DPP-4 inhibitor vildagliptin (10 mg/kg) in STZ-induced diabetic rats suppresses plasma TNF-α concentrations and inhibits nitric oxide concentrations in the serum (Akarte et al., 2012). Exendin-4 (10 nM) promotes the expression of serine protease inhibitor-9, thus underlying the survival capability of cells against the attack of immune cells such as natural killer cells and cytotoxic T cells in human islets (Cechin et al., 2012).

In the brain, GLP-1 treatment has a preventive effect on the progression of Alzheimer’s disease pathology in rats (Iwai et al., 2014; Solmaz et al., 2015). Exenatide-4 (20 µg/kg/day). GLP-1 treatment suppressed the level of TNF-α in an Alzheimer’s disease animal model brain induced by injection of STZ (Solmaz et al., 2015). Additionally, GLP-1 (50 nM) protected against synaptic dysfunction in the rat hippocampus induced by injection of lipopolysaccharide (LPS) (Iwai et al., 2014). In an Alzheimer’s disease mouse model, liraglutide (25 nmol/kg/day) treatment attenuated the neuroinflammation response in the cortex (McClean et al., 2011). In addition, the Alzheimer’s disease APPSWE/PS1ΔE9 mouse model showed a reduction of neuroinflammation upon liraglutide treatment (Holscher, 2014). Exendin-4 treatment (0.5 µg/kg) resulted in a reduction of pro-inflammatory cytokines such as TNF-α and IL-1β in CSF and in hippocampal and cortical brain areas (Ventorp et al., 2017).

Thus, GLP-1 can improve responses to neuroinflammation, and controls the production of cytokines in the brain. This function of GLP-1 under an inflammatory condition shows the therapeutic possibility of GLP-1 in the depressive brain accompanied by neuroinflammation.

### Neurotransmitter Imbalance in Depression and Its Relation to GLP-1

Neurotransmitters play a cardinal role in the brain and contribute to the regulation of behavior (Lener et al., 2017). Depression is typically thought to arise from a neurotransmitter imbalance (**Figure 2**) (Lener et al., 2017). Previous studies have demonstrated that the deficiency of monoaminergic neurotransmitters, such as 5-HT, norepinephrine (NE), and dopamine (DA), aggravates depressive behaviors and can be used as a diagnostic index for depression (Massart et al., 2012; Hamon and Blier, 2013). Serotonin is critical for mood processing and emotional regulation in brain areas such as the amygdala and hippocampus (Hervas et al., 2000; McKie et al., 2005), and may be essential for ameliorating depressive behaviors. Clinically depressed patients reportedly have impaired glutamatergic and hyperactive acetylcholine systems, whereas they have a dramatically suppressed gamma-aminobutyric acid (GABA) system (Pytka et al., 2016; Murrough et al., 2017). A previous brain magnetic resonance spectroscopy imaging study demonstrated that the levels of GABA and glutamate cycling was abnormal in patients with depression when compared to those in the normal brain (Sanacora et al., 2008). Yuen et al. demonstrated that chronic stress disrupts glutamate transmission in the PFC and cognition-related brain regions (Yuen et al., 2012). A number of other studies have also suggested that chronic stress causes atrophy of the PFC and hippocampus owing to glucocorticoid imbalance (Eisch and Petrik, 2012; McEwen et al., 2012).

Brain-derived neurotrophic factor (BDNF) is important for neurogenesis, and various depressive symptoms arise from decreased BDNF levels (Sahay and Hen, 2007). A previous study reported that anti-depressant therapy can increase the level of BDNF and subsequently boost neurogenesis, attenuate neuronal apoptosis in the depressive brain, and ultimately improve depressive mood (Sahay and Hen, 2007). Furthermore, recovery of BDNF levels results in the improvement of neuroplasticity and reduction of neuronal apoptosis (Alves et al., 2017; Kraus et al., 2017). Decreased BDNF in the hippocampus and PFC has been noted in depressed patients (Krishnan and Nestler, 2008; Duman and Voleti, 2012). Anti-depressants, which increase BDNF levels, lead to improvements in depressive behavior in BDNF-deletion mutant mice (Taliaz et al., 2010; Duman and Voleti, 2012; Yu et al., 2012). Some studies have reported that depression caused by chronic stress disrupts the BDNF–tropomyosin related kinase B (TrkB) receptor signaling pathway in the PFC and hippocampus, and ultimately contributes to impaired synaptic maturation, synaptic protein synthesis, and glutamate receptor cycling (Collingridge et al., 2010; Duric et al., 2010; Hoeffer and Klann, 2010; Christoffel et al., 2011; Duman and Voleti, 2012).

Therefore, because neurotransmitter imbalance contributes to depressive behaviors in patients with depression as described above, the appropriate modulation of neurotransmitters in the brain is key to treating depression (**Figure 2**).

A previous study reported that GLP-1Rs are observed in brain regions related to energy balance and regulation of moods, such as the amygdala, hippocampus, and dorsal raphe nucleus (Merchenthaler et al., 1999). Some studies have also shown the presence of GLP-1Rs in mood-related brain regions and demonstrated its powerful role in emotional processing within the CNS (Merchenthaler et al., 1999; Sharma et al., 2015).

Serotonin is produced by specific neurons localized in raphe nuclei in the brainstem (Llewellyn-Smith et al., 2013). These neurons innervate the amygdala (Vertes, 1991) and the hippocampus (McQuade and Sharp, 1997). The endogenous ligands of GLP-1-producing neurons were found to innervate the brainstem raphe nuclei (Llewellyn-Smith et al., 2013). Another study reported that injection of the GLP-1 agonist exendin-4 influenced the dopamine system and reduced food-related reward behavior in rats (Hayes and Schmidt, 2016). In patients with depression, the reward learning process is disrupted, and impaired reward learning is thought to lead to depressive mood and behavior (Fletcher and Reback, 2015). Dopamine regulates the reward learning system by stimulating the mesolimbic circle (Steinberg et al., 2013; Lietzau et al., 2020).

The exendin-4 treatment has been shown to reduce cocaine self-administration, suggesting that the GLP-1 system may be a novel target of drug addiction (Sorensen et al., 2015). Dopamine as a neurotransmitter has also been shown to reduce depressive-like behaviors such as anxiety (Dunlop and Nemeroff, 2007; Tye et al., 2013; Mohammadi et al., 2015; Lietzau et al., 2020). Recent studies have demonstrated that the GLP-1 agonist, liraglutide, exerts an antipsychotic effect in a mouse model of psychosis (Dixit et al., 2013). GLP-1 can elevate the turnover of dopamine in the brain amygdala through dopamine receptor 2 signaling (Anderberg et al., 2014; Lietzau et al., 2020). Furthermore, GLP-1Rs have been shown to be able to stimulate the depolarization-evoked release of other neurotransmitters, such as GABA and glutamate, as well as of serotonin and dopamine, in the cortex and hippocampus (Rebosio et al., 2018).

These studies indicate that GLP-1 modulates the release of several neurotransmitters including serotonin, dopamine, GABA, and glutamate, which may regulate depressive-like behaviors. Modulation of neurotransmitter secretion by GLP-1 may be another effective solution for alleviating the effects of depression.

### Neurogenesis in Depression and Its Relation to GLP-1

In the brain, neurogenesis is a critical repair response against stress and age (Dranovsky et al., 2011). New neurons generated through the neurogenesis process are produced from neural progenitors mainly located in the subgranular zone of the dentate gyrus (Dranovsky et al., 2011). Neurogenesis repairs the damaged brain and improves cognition (Schmidt-Hieber et al., 2004). The depressive brain has been shown to exhibit a decrease in hippocampal neurogenesis, and this reduction is recovered by antidepressant drug treatment (Tanti and Belzung, 2013). Some brain imaging studies have demonstrated that depressed patients show neuronal atrophy and decreased volume of the cortex and limbic lobe regions, including the PFC and hippocampus. These regions are known to regulate emotion and cognition, and pathological problems and impaired cognitive function are observed in depressive conditions (Price and Drevets, 2010; MacQueen and Frodl, 2011).

Patients with recurrent depression exhibit reduced hippocampal volume and hippocampal atrophy in the brain when compared to healthy controls (**Figure 2**) (Bremner et al., 2000). Moreover, the decrease in hippocampal volume is significantly related to the total duration of depressive episodes (Sheline et al., 1999). Other studies have reported that a reduction in hippocampal neurogenesis leads to an increase in anxiety symptoms and the onset of depression (Hanson et al., 2011; Eisch and Petrik, 2012; Moylan et al., 2013; Smitha et al., 2014). Increased hippocampal neurogenesis influences anxiety and depressive behavior through the HPA axis and is considered a promising strategy to promote antidepressant-like effects (Hill et al., 2015).

Calcium signaling resulting from GABA receptor activation has been found to promote neuronal differentiation and control the synaptic integration of neuronal precursor cells (Ge et al., 2006). A serotonin 5-HT_2A_ receptor antagonist was shown to reduce neuronal proliferation, and chronic administration of a 5-HT_1A_ receptor agonist increased neuronal proliferation (Jha et al., 2008). With this perspective, the regulation of neurotransmitter secretion may be considerably linked with the activation of neurogenesis in the depressive brain. Moreover, chemokines are important regulators of neuronal proliferation and neural progenitor cell differentiation (Tran et al., 2007; Miller et al., 2008). As such, cytokine and chemokine dysregulation likely contribute to impaired neurogenesis in the depressive brain. Thus, we expect that the promotion of neurogenesis in the brain may be a key strategy to cure depression.

The GLP-1R agonist exendin-4 was shown to promote neurogenesis in the subventricular zone region in a neurodegenerative disease rodent model (Bertilsson et al., 2008). Interestingly, several studies have demonstrated that exendin-4 has neurotrophic and neuroprotective effects and that it enhances neurogenesis and neural proliferation (Li et al., 2009; Holscher, 2014; Sango and Utsunomiya, 2015). Furthermore, Isacson et al. demonstrated that GLP-1 agonist administration could lead to an increase in neurogenesis in the hippocampus dentate gyrus region, and demonstrated an improvement in mood and cognitive function through the swim test in adult rodents (Isacson et al., 2011). Moreover, GLP-1 has been reported to enhance synaptic plasticity and synaptic formation in the hippocampus (Cai et al., 2014). The increase of hippocampal neurogenesis is essential for improved cognitive function induced by environmental factors such as exercise (Petrik et al., 2012; Klempin et al., 2013).

Previous studies have demonstrated that neurogenesis influences emotional regulation in adult mice (Deng et al., 2009), and that enhanced neurogenesis in the hippocampus acts as an antidepressant for increasing serotonin receptor activity (Wang et al., 2008; David et al., 2009; Kondo et al., 2015). Moreover, hippocampal neurogenesis reduces neuronal damage by replacing lost neurons, which appears to improve depressive-like behavior (Zhang et al., 2008). It has been demonstrated that the administration of GLP-1 analogs, including liraglutide and exendin-4, promotes neurogenesis and neural progenitor cell proliferation in the hippocampus of rodent models (Darsalia et al., 2012; McGovern et al., 2012; Porter et al., 2013). It has also been reported that liraglutide increases neural stem cell proliferation and neuronal differentiation in the APP/PS1 neurodegenerative disease model (Parthsarathy and Holscher, 2013a). Moreover, a GLP-1 analog, (Val(8))GLP-1-Glu-PAL, increased hippocampal neurogenesis and cell proliferation in obese mice fed a high-fat diet (Lennox et al., 2013). Further, the expression of neurogenesis-related proteins was promoted by the activation of the cAMP/PKA and Akt/GSK3 signaling pathways through the stimulation of GLP-1R in mice (Wang et al., 2018). From *in vitro* studies using human SH-SY5Y neuronal cells, it was demonstrated that cell proliferation is increased with GLP-1 treatment (Li et al., 2010; Salcedo et al., 2012).

Some studies demonstrated that the administration of antidepressants leads to an improvement in depressive mood through the increase of hippocampal neurogenesis and the enhancement of synaptic connectivity (Duman et al., 2001; Toni et al., 2007; Boldrini et al., 2009; Denny et al., 2012). Several studies have demonstrated that increasing neurogenesis in the brain is an effective therapeutic approach for reducing depressive-like behaviors (Anacker et al., 2011; Mendez-David et al., 2013; Weina et al., 2018). A previous study indicated that treatment with the GLP-1 agonist liraglutide (subcutaneously at a dose gradually titrated from 0.6 to 3 mg) in bipolar and mood disorder depression patients could result in an improvement in cognitive function and an alleviation of depressive pathological symptoms (Cuomo et al., 2018).

Considering this evidence, increasing neurogenesis in the brain through the GLP-1 pathway may provide a novel approach for improving depressive-like behavior in patients with depression.

### Synaptic Dysfunction and Memory Loss in Depression and Its Relation to GLP-1

In patients with depression, neural circuits are impaired as a result of aberrant communication between neurons (Williams, 2016). Functional brain imaging studies have reported a decrease in synaptic connectivity and neuronal circuitry in the PFC and hippocampus in depressed patients (Perlman et al., 2012; Zeng et al., 2012). Moreover, postmortem brains of depressed patients are reduced in size and show loss of pyramidal neurons (Rajkowska et al., 1999), GABAergic interneurons, and glia in the PFC (Rajkowska et al., 2007). Several neuronal morphological studies also reported a reduction in synapse number and synaptic signaling proteins in the PFC of depressed patients through electron microscopy (Kang et al., 2012). Reduced glutamate receptors, presynaptic neurotransmitter proteins, and postsynaptic functional proteins in the PFC and hippocampus were also observed (**Figure 2**) (Zhao et al., 2012; Duric et al., 2013).

Chronic unpredictable stress, considered a model of depression, results in a reduction in dendritic lengths and branching of apical dendrites, the loss of functional synaptic spines, and the reduction of dendritic complexity in PFC neurons and in the hippocampal CA3 pyramidal neuronal cell layer (McEwen et al., 2012; Morrison and Baxter, 2012). Furthermore, chronic stress aggravates neuronal damage in the amygdala and nucleus accumbens and subsequently disrupts the motivation and reward system (Duman and Voleti, 2012). Another study reported that depression caused by chronic stress blocks glutamate signaling and synaptic transmission, and this ultimately leads to cognitive dysfunction (Yuen et al., 2012). Moreover, depressed patients show a reduction in synaptic density proteins in response to stress (Kang et al., 2012). Depression reportedly increases the negative regulation of ERK signaling associated with synaptic plasticity (Duric et al., 2010) and inhibits downstream signaling of BDNF and neuritin that are essential for normal synaptic function (Son et al., 2012).

Glycogen synthase kinase 3 (GSK3) has been shown to regulate synaptic homeostasis in the brain (Collingridge et al., 2010). Many researchers have demonstrated that depressed patients have excessive activation of GSK3-deconsolidation, which leads to a reduction in synaptic spines (Li and Jope, 2010; Wilkinson et al., 2011). Previous studies have shown that synaptic plasticity, synaptic transmission, and long-term potentiation (LTP) are dramatically attenuated in the depressive brain (Karpova et al., 2011; Bath et al., 2012). In dendrites and cell bodies of the neuron, activation of the mammalian target of the rapamycin (mTOR) signaling pathway accelerates long-term synaptogenesis (Hoeffer and Klann, 2010). In the depressive brain, activation of mTOR is reduced, which subsequently decreases the release of BDNF and the synthesis of synaptic proteins (Jourdi et al., 2009).

Most patients with depression show mood disturbances, as well as general cognitive impairment, which may explain the two-fold greater risk of dementia development seen in such patients (Bulbena and Berrios, 1986; Kohler et al., 2010; Byers et al., 2012; Tsai and Rosenheck, 2016). A neuroimaging study suggested that depression strongly leads to memory dysfunction given that morphological changes in the brain of depressed patients, such as atrophy and abnormal alteration of the frontal cortex, thalamus, and hippocampus, is related to cognitive impairment (Zhang et al., 2016).

Based on these reports, the impairment of synaptic function and synaptic transmission and the loss of synaptic density proteins are widely observed in the depressive brain (**Figure 2**). Synaptic dysfunction may lead to memory dysfunction in patients with depression. Therefore, improving synaptic function is an important component for treating depressive neuropathology.

One study reported that GLP-1 is negatively correlated with body mass index (BMI) and that it appears to increase dentate gyrus neurogenesis based on immunostaining images (Coplan et al., 2014). Recent research has demonstrated that liraglutide promotes neurite outgrowth of cortical neurons in severe oxidative stress conditions through Wnt signaling (He et al., 2018). Furthermore, another study has highlighted the role of liraglutide in preventing depressive-like behaviors by enhancing hippocampal neuron synaptic plasticity (Weina et al., 2018). Gault et al. demonstrated that oral administration of the DPP inhibitor sitagliptin (50 mg/kg) can enhance cognitive function and protects neurons against oxidative stress in high fat-fed mice (Gault et al., 2015). One *in vitro* study reported that exendin-4 increases the number of neurites, promotes neuronal outgrowth, and considerably increases neurite length (Luciani et al., 2010).

It was previously shown that the GLP-1 receptor can control neuronal function in the rat hippocampus by enhancing GABAA signaling through presynaptic and postsynaptic mechanisms (Korol et al., 2015). Previous studies reported that the administration of the GLP-1R agonist exendin-4 (25 nmol/kg, twice daily) inhibits learning and memory formation through the suppression of synaptic plasticity in the hippocampus of a high fat diet mouse model (Gault et al., 2010; Lennox et al., 2014).

LTP results from the synchronous activity of neurons and is widely considered an indicator of memory formation. One study demonstrated that GLP-1 can activate LTP in the brain and ameliorate cognitive dysfunction in a neurodegenerative disorder model (McClean et al., 2011). Another study showed that GLP-1R knockout mice have impaired LTP when compared to control animals (Abbas et al., 2009). Overexpression of GLP-1Rs in the mouse brain leads to enhanced learning, as measured with the Morris water maze and learning performance tests (During et al., 2003). Interestingly, a recent study showed that GLP-1 and exendin-4 induced anxiety-like behaviors in mice (Anderberg et al., 2016). The stimulation of GLP-1Rs alters serotonin signaling in the amygdala (Anderberg et al., 2016). Importantly, chronic administration of exendin-4 significantly reduces depression-like behavior (Anderberg et al., 2016).

These previous studies together suggest that GLP-1 may improve memory and cognitive function in patients with depression by enhancing synaptic function and neuronal signal transmission in the brain.

## Conclusions

In this review, we have presented four cardinal points about the roles of GLP-1 in the depressive brain. First, we summarized significant evidence pointing to the relationship between neuroinflammation and GLP-1 administration. GLP-1 appears to attenuate the process of neuroinflammation and protects neurons and glia under oxidative stress conditions in the depressive brain. Second, we described the role of GLP-1 in neurotransmitter homeostasis in the depressive brain in which GLP-1 can improve neurotransmitter balance. In the depressive brain, the secretion of diverse neurotransmitters is not stable compared to that in the normal brain. GLP-1 is a useful therapeutic modulator of depression, suggesting that abnormal alteration of neurotransmitter levels in the depressive brain results in mood impairment and cognitive decline. Third, we reviewed the role GLP-1 in promoting neuronal differentiation and neural stem cell proliferation in the depressive brain. GLP-1 is a promising target for the treatment of depression because impaired neurogenesis ability and the reduction of neuronal differentiation leads to multiple depressive pathological symptoms. Finally, we summarized studies showing that GLP-1 can enhance cognitive decline by improving synaptic function in the depressive brain. GLP-1 ameliorates synaptic dysfunction in the depressive brain and subsequently leads to the enhancement of cognitive function. Hence, GLP-1 may be key to improving cognitive decline in patients with depression.

Taken together, we have highlighted GLP-1 as a novel therapeutic marker for identifying and treating the neuropathology of depression. Furthermore, we emphasize the necessity of further studies concerning the mechanisms and function of GLP-1 in the depressive brain. Similar to the use of GLP-1 analogs in diabetic patients, we believe that GLP-1 may be used for patients with depression in the near future.

## Author Contributions

Y-KK contributed to the writing of the text and provided the table and figures. OK and JS wrote and revised the manuscript. OK provided financial support for the study. JS finalized the revised manuscript.

## Funding

This research was supported by the Basic Science Research Program through the National Research Foundation of Korea (NRF) funded by the Ministry of Education (grant number: NRF-2019R1F1A1054111 (JS), NRF-2019R1I1A3A01058861 (OK), and NRF-2018R1A2B6001104 (Y-KK).

## Conflict of Interest

The authors declare that the research was conducted in the absence of any commercial or financial relationships that could be construed as a potential conflict of interest.

## References

[B1] Abbas T. Faivre E. Holscher C. (2009). Impairment of synaptic plasticity and memory formation in GLP-1 receptor KO mice: Interaction between type 2 diabetes and Alzheimer’s disease. Behav. Brain Res. 205, 265–271. 10.1016/j.bbr.2009.06.035 19573562

[B2] Akarte A. S. Srinivasan B. P. Gandhi S. Sole S. (2012). Chronic DPP-IV inhibition with PKF-275-055 attenuates inflammation and improves gene expressions responsible for insulin secretion in streptozotocin induced diabetic rats. Eur. J. Pharm. Sci. 47, 456–463. 10.1016/j.ejps.2012.07.003 22800967

[B3] Akimoto H. Tezuka K. Nishida Y. Nakayama T. Takahashi Y. Asai S. (2019). Association between use of oral hypoglycemic agents in Japanese patients with type 2 diabetes mellitus and risk of depression: A retrospective cohort study. Pharmacol. Res. Perspect. 7, e00536. 10.1002/prp2.536 31768258 PMC6868652

[B4] Alhadeff A. L. Rupprecht L. E. Hayes M. R. (2012). GLP-1 neurons in the nucleus of the solitary tract project directly to the ventral tegmental area and nucleus accumbens to control for food intake. Endocrinology 153, 647–658. 10.1210/en.2011-1443 22128031 PMC3275387

[B5] Al-Harbi K. S. (2012). Treatment-resistant depression: therapeutic trends, challenges, and future directions. Patient Prefer. Adherence 6, 369–388. 10.2147/PPA.S29716 22654508 PMC3363299

[B6] Alves N. D. Correia J. S. Patricio P. Mateus-Pinheiro A. Machado-Santos A. R. Loureiro-Campos E. (2017). Adult hippocampal neuroplasticity triggers susceptibility to recurrent depression. Transl. Psychiatry 7, e1058. 10.1038/tp.2017.29 28291258 PMC5416672

[B7] Anacker C. Zunszain P. A. Cattaneo A. Carvalho L. A. Garabedian M. J. Thuret S. (2011). Antidepressants increase human hippocampal neurogenesis by activating the glucocorticoid receptor. Mol. Psychiatry 16, 738–750. 10.1038/mp.2011.26 21483429 PMC3121947

[B8] Anderberg R. H. Anefors C. Bergquist F. Nissbrandt H. Skibicka K. P. (2014). Dopamine signaling in the amygdala, increased by food ingestion and GLP-1, regulates feeding behavior. Physiol. Behav. 136, 135–144. 10.1016/j.physbeh.2014.02.026 24560840

[B9] Anderberg R. H. Richard J. E. Hansson C. Nissbrandt H. Bergquist F. Skibicka K. P. (2016). GLP-1 is both anxiogenic and antidepressant; divergent effects of acute and chronic GLP-1 on emotionality. Psychoneuroendocrinology 65, 54–66. 10.1016/j.psyneuen.2015.11.021 26724568

[B10] Astrup A. Rossner S. Van Gaal L. Rissanen A. Niskanen L. Al Hakim M. (2009). Effects of liraglutide in the treatment of obesity: a randomised, double-blind, placebo-controlled study. Lancet 374, 1606–1616. 10.1016/S0140-6736(09)61375-1 19853906

[B11] Athauda D. Foltynie T. (2016). The glucagon-like peptide 1 (GLP) receptor as a therapeutic target in Parkinson’s disease: mechanisms of action. Drug Discov. Today 21, 802–818. 10.1016/j.drudis.2016.01.013 26851597

[B12] Augestad I. L. Pintana H. Larsson M. Krizhanovskii C. Nystrom T. Klein T. (2020). The Regulation of Glycemia in the Recovery Phase After Stroke Counteracts the Detrimental Effect of Obesity-Induced Type 2 Diabetes on Neurological Recovery. Diabetes db200095. 10.2337/db20-0095 32540876

[B13] Bath K. G. Jing D. Q. Dincheva I. Neeb C. C. Pattwell S. S. Chao M. V. (2012). BDNF Val66Met impairs fluoxetine-induced enhancement of adult hippocampus plasticity. Neuropsychopharmacology 37, 1297–1304. 10.1038/npp.2011.318 22218094 PMC3306891

[B14] Belmaker R. H. Agam G. (2008). Major depressive disorder. N Engl. J. Med. 358, 55–68. 10.1056/NEJMra073096 18172175

[B15] Bertilsson G. Patrone C. Zachrisson O. Andersson A. Dannaeus K. Heidrich J. (2008). Peptide hormone exendin-4 stimulates subventricular zone neurogenesis in the adult rodent brain and induces recovery in an animal model of Parkinson’s disease. J. Neurosci. Res. 86, 326–338. 10.1002/jnr.21483 17803225

[B16] Black C. Miller B. J. (2015). Meta-Analysis of Cytokines and Chemokines in Suicidality: Distinguishing Suicidal Versus Nonsuicidal Patients. Biol. Psychiatry 78, 28–37. 10.1016/j.biopsych.2014.10.014 25541493

[B17] Boldrini M. Underwood M. D. Hen R. Rosoklija G. B. Dwork A. J. John Mann J. (2009). Antidepressants increase neural progenitor cells in the human hippocampus. Neuropsychopharmacology 34, 2376–2389. 10.1038/npp.2009.75 19606083 PMC2743790

[B18] Bremner J. D. Narayan M. Anderson E. R. Staib L. H. Miller H. L. Charney D. S. (2000). Hippocampal volume reduction in major depression. Am. J. Psychiatry 157, 115–118. 10.1176/ajp.157.1.115 10618023

[B19] Bulbena A. Berrios G. E. (1986). Pseudodementia: facts and figures. Br. J. Psychiatry 148, 87–94. 10.1192/bjp.148.1.87 3955324

[B20] Bullock B. P. Heller R. S. Habener J. F. (1996). Tissue distribution of messenger ribonucleic acid encoding the rat glucagon-like peptide-1 receptor. Endocrinology 137, 2968–2978. 10.1210/endo.137.7.8770921 8770921

[B21] Byers A. L. Covinsky K. E. Barnes D. E. Yaffe K. (2012). Dysthymia and depression increase risk of dementia and mortality among older veterans. Am. J. Geriatr. Psychiatry 20, 664–672. 10.1097/JGP.0b013e31822001c1 21597358 PMC3229643

[B22] Cabou C. Burcelin R. (2011). GLP-1, the gut-brain, and brain-periphery axes. Rev. Diabetes Stud. 8, 418–431. 10.1900/RDS.2011.8.418 PMC3280675 22262078

[B23] Cai H. Y. Holscher C. Yue X. H. Zhang S. X. Wang X. H. Qiao F. (2014). Lixisenatide rescues spatial memory and synaptic plasticity from amyloid beta protein-induced impairments in rats. Neuroscience 277, 6–13. 10.1016/j.neuroscience.2014.02.022 24583037

[B24] Calsolaro V. Edison P. (2015). Novel GLP-1 (Glucagon-Like Peptide-1) Analogues and Insulin in the Treatment for Alzheimer’s Disease and Other Neurodegenerative Diseases. CNS Drugs 29, 1023–1039. 10.1007/s40263-015-0301-8 26666230

[B25] Campbell J. E. Drucker D. J. (2013). Pharmacology, physiology, and mechanisms of incretin hormone action. Cell Metab. 17, 819–837. 10.1016/j.cmet.2013.04.008 23684623

[B26] Candeias E. M. Sebastiao I. C. Cardoso S. M. Correia S. C. Carvalho C. I. Placido A. I. (2015). Gut-brain connection: The neuroprotective effects of the anti-diabetic drug liraglutide. World J. Diabetes 6, 807–827. 10.4239/wjd.v6.i6.807 26131323 PMC4478577

[B27] Carvalho L. A. Torre J. P. Papadopoulos A. S. Poon L. Juruena M. F. Markopoulou K. (2013). Lack of clinical therapeutic benefit of antidepressants is associated overall activation of the inflammatory system. J. Affect. Disord. 148, 136–140. 10.1016/j.jad.2012.10.036 23200297

[B28] Cechin S. R. Perez-Alvarez I. Fenjves E. Molano R. D. Pileggi A. Berggren P. O. (2012). Anti-inflammatory properties of exenatide in human pancreatic islets. Cell Transplant. 21, 633–648. 10.3727/096368911X576027 21669040

[B29] Christoffel D. J. Golden S. A. Russo S. J. (2011). Structural and synaptic plasticity in stress-related disorders. Rev. Neurosci. 22, 535–549. 10.1515/RNS.2011.044 21967517 PMC3212803

[B30] Collingridge G. L. Peineau S. Howland J. G. Wang Y. T. (2010). Long-term depression in the CNS. Nat. Rev. Neurosci. 11, 459–473. 10.1038/nrn2867 20559335

[B31] Collins S. M. Bercik P. (2009). The relationship between intestinal microbiota and the central nervous system in normal gastrointestinal function and disease. Gastroenterology 136, 2003–2014. 10.1053/j.gastro.2009.01.075 19457424

[B32] Coplan J. D. Syed S. Perera T. D. Fulton S. L. Banerji M. A. Dwork A. J. (2014). Glucagon-like peptide-1 as predictor of body mass index and dentate gyrus neurogenesis: neuroplasticity and the metabolic milieu. Neural Plast. 2014, 917981. 10.1155/2014/917981 25506432 PMC4259073

[B33] Cork S. C. Richards J. E. Holt M. K. Gribble F. M. Reimann F. Trapp S. (2015). Distribution and characterisation of Glucagon-like peptide-1 receptor expressing cells in the mouse brain. Mol. Metab. 4, 718–731. 10.1016/j.molmet.2015.07.008 26500843 PMC4588458

[B34] Crane J. McGowan B. (2016). The GLP-1 agonist, liraglutide, as a pharmacotherapy for obesity. Ther. Adv. Chronic Dis. 7, 92–107. 10.1177/2040622315620180 26977279 PMC4772342

[B35] Cuomo A. Bolognesi S. Goracci A. Ciuoli C. Beccarini Crescenzi B. Maina G. (2018). Feasibility, Adherence and Efficacy of Liraglutide Treatment in a Sample of Individuals With Mood Disorders and Obesity. Front. Psychiatry 9, 784. 10.3389/fpsyt.2018.00784 30728788 PMC6351474

[B36] Darsalia V. Mansouri S. Ortsater H. Olverling A. Nozadze N. Kappe C. (2012). Glucagon-like peptide-1 receptor activation reduces ischaemic brain damage following stroke in Type 2 diabetic rats. Clin. Sci. (Lond.) 122, 473–483. 10.1042/CS20110374 22150224 PMC3268352

[B37] Darsalia V. Hua S. Larsson M. Mallard C. Nathanson D. Nystrom T. (2014a). Exendin-4 reduces ischemic brain injury in normal and aged type 2 diabetic mice and promotes microglial M2 polarization. PLoS One 9, e103114. 10.1371/journal.pone.0103114 25101679 PMC4125154

[B38] Darsalia V. Olverling A. Larsson M. Mansouri S. Nathanson D. Nystrom T. (2014b). Linagliptin enhances neural stem cell proliferation after stroke in type 2 diabetic mice. Regul. Pept. 190-191, 25–31. 10.1016/j.regpep.2014.05.001 24821550

[B39] David D. J. Samuels B. A. Rainer Q. Wang J. W. Marsteller D. Mendez I. (2009). Neurogenesis-dependent and -independent effects of fluoxetine in an animal model of anxiety/depression. Neuron 62, 479–493. 10.1016/j.neuron.2009.04.017 19477151 PMC2759281

[B40] de Miranda A. S. Zhang C. J. Katsumoto A. Teixeira A. L. (2017). Hippocampal adult neurogenesis: Does the immune system matter? J. Neurol. Sci. 372, 482–495. 10.1016/j.jns.2016.10.052 27838002

[B41] Deng W. Saxe M. D. Gallina I. S. Gage F. H. (2009). Adult-born hippocampal dentate granule cells undergoing maturation modulate learning and memory in the brain. J. Neurosci. 29, 13532–13542. 10.1523/JNEUROSCI.3362-09.2009 19864566 PMC2787190

[B42] Denny C. A. Burghardt N. S. Schachter D. M. Hen R. Drew M. R. (2012). 4- to 6-week-old adult-born hippocampal neurons influence novelty-evoked exploration and contextual fear conditioning. Hippocampus 22, 1188–1201. 10.1002/hipo.20964 21739523 PMC3193906

[B43] Disease G. B. D. Injury I. Prevalence C. (2016). Global, regional, and national incidence, prevalence, and years lived with disability for 310 diseases and injuries 1990-2015: a systematic analysis for the Global Burden of Disease Study 2015. Lancet 388, 1545–1602. 10.1016/S0140-6736(16)31678-6 27733282 PMC5055577

[B44] Dixit T. S. Sharma A. N. Lucot J. B. Elased K. M. (2013). Antipsychotic-like effect of GLP-1 agonist liraglutide but not DPP-IV inhibitor sitagliptin in mouse model for psychosis. Physiol. Behav. 114-115, 38–41. 10.1016/j.physbeh.2013.03.008 23523479

[B45] do Prado C. H. Narahari T. Holland F. H. Lee H. N. Murthy S. K. Brenhouse H. C. (2016). Effects of early adolescent environmental enrichment on cognitive dysfunction, prefrontal cortex development, and inflammatory cytokines after early life stress. Dev. Psychobiol. 58, 482–491. 10.1002/dev.21390 26688108 PMC12878819

[B46] Dobrian A. D. Ma Q. Lindsay J. W. Leone K. A. Ma K. Coben J. (2011). Dipeptidyl peptidase IV inhibitor sitagliptin reduces local inflammation in adipose tissue and in pancreatic islets of obese mice. Am. J. Physiol. Endocrinol. Metab. 300, E410–E421. 10.1152/ajpendo.00463.2010 21081706 PMC3043624

[B47] Dranovsky A. Picchini A. M. Moadel T. Sisti A. C. Yamada A. Kimura S. (2011). Experience dictates stem cell fate in the adult hippocampus. Neuron 70, 908–923. 10.1016/j.neuron.2011.05.022 21658584 PMC3124009

[B48] Drucker D. J. Nauck M. A. (2006). The incretin system: glucagon-like peptide-1 receptor agonists and dipeptidyl peptidase-4 inhibitors in type 2 diabetes. Lancet 368, 1696–1705. 10.1016/S0140-6736(06)69705-5 17098089

[B49] Duarte A. I. Candeias E. Correia S. C. Santos R. X. Carvalho C. Cardoso S. (2013). Crosstalk between diabetes and brain: glucagon-like peptide-1 mimetics as a promising therapy against neurodegeneration. Biochim. Biophys. Acta 1832, 527–541. 10.1016/j.bbadis.2013.01.008 23314196

[B50] Duman R. S. Voleti B. (2012). Signaling pathways underlying the pathophysiology and treatment of depression: novel mechanisms for rapid-acting agents. Trends Neurosci. 35, 47–56. 10.1016/j.tins.2011.11.004 22217452 PMC3278537

[B51] Duman R. S. Nakagawa S. Malberg J. (2001). Regulation of adult neurogenesis by antidepressant treatment. Neuropsychopharmacology 25, 836–844. 10.1016/S0893-133X(01)00358-X 11750177

[B52] Dunlop B. W. Nemeroff C. B. (2007). The role of dopamine in the pathophysiology of depression. Arch. Gen. Psychiatry 64, 327–337. 10.1001/archpsyc.64.3.327 17339521

[B53] Duric V. Banasr M. Licznerski P. Schmidt H. D. Stockmeier C. A. Simen A. A. (2010). A negative regulator of MAP kinase causes depressive behavior. Nat. Med. 16, 1328–1332. 10.1038/nm.2219 20953200 PMC3066515

[B54] Duric V. Banasr M. Stockmeier C. A. Simen A. A. Newton S. S. Overholser J. C. (2013). Altered expression of synapse and glutamate related genes in post-mortem hippocampus of depressed subjects. Int. J. Neuropsychopharmacol. 16, 69–82. 10.1017/S1461145712000016 22339950 PMC3414647

[B55] During M. J. Cao L. Zuzga D. S. Francis J. S. Fitzsimons H. L. Jiao X. (2003). Glucagon-like peptide-1 receptor is involved in learning and neuroprotection. Nat. Med. 9, 1173–1179. 10.1038/nm919 12925848

[B56] Edholm T. Degerblad M. Gryback P. Hilsted L. Holst J. J. Jacobsson H. (2010). Differential incretin effects of GIP and GLP-1 on gastric emptying, appetite, and insulin-glucose homeostasis. Neurogastroenterol. Motil. 22, 1191–1200, e1315. 10.1111/j.1365-2982.2010.01554.x 20584260

[B57] Eisch A. J. Petrik D. (2012). Depression and hippocampal neurogenesis: a road to remission? Science 338, 72–75. 10.1126/science.1222941 23042885 PMC3756889

[B58] Evrensel A. Ceylan M. E. (2015). The Gut-Brain Axis: The Missing Link in Depression. Clin. Psychopharmacol. Neurosci. 13, 239–244. 10.9758/cpn.2015.13.3.239 26598580 PMC4662178

[B59] Felger J. C. Treadway M. T. (2017). Inflammation Effects on Motivation and Motor Activity: Role of Dopamine. Neuropsychopharmacology 42, 216–241. 10.1038/npp.2016.143 27480574 PMC5143486

[B60] Fletcher J. B. Reback C. J. (2015). Depression mediates and moderates effects of methamphetamine use on sexual risk taking among treatment-seeking gay and bisexual men. Health Psychol. 34, 865–869. 10.1037/hea0000207 25581704 PMC7685202

[B61] Forsythe P. Sudo N. Dinan T. Taylor V. H. Bienenstock J. (2010). Mood and gut feelings. Brain Behav. Immun. 24, 9–16. 10.1016/j.bbi.2009.05.058 19481599

[B62] Gault V. A. Porter W. D. Flatt P. R. Holscher C. (2010). Actions of exendin-4 therapy on cognitive function and hippocampal synaptic plasticity in mice fed a high-fat diet. Int. J. Obes. (Lond.) 34, 1341–1344. 10.1038/ijo.2010.59 20351729

[B63] Gault V. A. Lennox R. Flatt P. R. (2015). Sitagliptin, a dipeptidyl peptidase-4 inhibitor, improves recognition memory, oxidative stress and hippocampal neurogenesis and upregulates key genes involved in cognitive decline. Diabetes Obes. Metab. 17, 403–413. 10.1111/dom.12432 25580570

[B64] Ge S. Goh E. L. Sailor K. A. Kitabatake Y. Ming G. L. Song H. (2006). GABA regulates synaptic integration of newly generated neurons in the adult brain. Nature 439, 589–593. 10.1038/nature04404 16341203 PMC1420640

[B65] Gerhard D. M. Wohleb E. S. Duman R. S. (2016). Emerging treatment mechanisms for depression: focus on glutamate and synaptic plasticity. Drug Discovery Today 21, 454–464. 10.1016/j.drudis.2016.01.016 26854424 PMC4803609

[B66] Gimeno D. Kivimaki M. Brunner E. J. Elovainio M. De Vogli R. Steptoe A. (2009). Associations of C-reactive protein and interleukin-6 with cognitive symptoms of depression: 12-year follow-up of the Whitehall II study. Psychol. Med. 39, 413–423. 10.1017/S0033291708003723 18533059 PMC2788760

[B67] Goldsmith D. R. Rapaport M. H. Miller B. J. (2016). A meta-analysis of blood cytokine network alterations in psychiatric patients: comparisons between schizophrenia, bipolar disorder and depression. Mol. Psychiatry 21, 1696–1709. 10.1038/mp.2016.3 26903267 PMC6056174

[B68] Haapakoski R. Ebmeier K. P. Alenius H. Kivimaki M. (2016). Innate and adaptive immunity in the development of depression: An update on current knowledge and technological advances. Prog. Neuropsychopharmacol. Biol. Psychiatry 66, 63–72. 10.1016/j.pnpbp.2015.11.012 26631274 PMC4736094

[B69] Habib A. M. Richards P. Rogers G. J. Reimann F. Gribble F. M. (2013). Co-localisation and secretion of glucagon-like peptide 1 and peptide YY from primary cultured human L cells. Diabetologia 56, 1413–1416. 10.1007/s00125-013-2887-z 23519462 PMC3648684

[B70] Hamilton A. Holscher C. (2009). Receptors for the incretin glucagon-like peptide-1 are expressed on neurons in the central nervous system. Neuroreport 20, 1161–1166. 10.1097/WNR.0b013e32832fbf14 19617854

[B71] Hamilton J. P. Farmer M. Fogelman P. Gotlib I. H. (2015). Depressive Rumination, the Default-Mode Network, and the Dark Matter of Clinical Neuroscience. Biol. Psychiatry 78, 224–230. 10.1016/j.biopsych.2015.02.020 25861700 PMC4524294

[B72] Hamon M. Blier P. (2013). Monoamine neurocircuitry in depression and strategies for new treatments. Prog. Neuropsychopharmacol. Biol. Psychiatry 45, 54–63. 10.1016/j.pnpbp.2013.04.009 23602950

[B73] Hansen H. H. Fabricius K. Barkholt P. Niehoff M. L. Morley J. E. Jelsing J. (2015). The GLP-1 Receptor Agonist Liraglutide Improves Memory Function and Increases Hippocampal CA1 Neuronal Numbers in a Senescence-Accelerated Mouse Model of Alzheimer’s Disease. J. Alzheimers Dis. 46, 877–888. 10.3233/JAD-143090 25869785 PMC4878312

[B74] Hanson N. D. Owens M. J. Nemeroff C. B. (2011). Depression, antidepressants, and neurogenesis: a critical reappraisal. Neuropsychopharmacology 36, 2589–2602. 10.1038/npp.2011.220 21937982 PMC3230505

[B75] Haroon E. Miller A. H. (2017). Inflammation Effects on Brain Glutamate in Depression: Mechanistic Considerations and Treatment Implications. Curr. Top. Behav. Neurosci. 31, 173–198. 10.1007/7854_2016_40 27830574

[B76] Hayes M. R. Schmidt H. D. (2016). GLP-1 influences food and drug reward. Curr. Opin. Behav. Sci. 9, 66–70. 10.1016/j.cobeha.2016.02.005 27066524 PMC4822543

[B77] He W. Tian X. Lv M. Wang H. (2018). Liraglutide Protects Neurite Outgrowth of Cortical Neurons Under Oxidative Stress though Activating the Wnt Pathway. J. Stroke Cerebrovasc. Dis. 27, 2696–2702. 10.1016/j.jstrokecerebrovasdis.2018.05.039 30042033

[B78] Heppner K. M. Kirigiti M. Secher A. Paulsen S. J. Buckingham R. Pyke C. (2015a). Expression and distribution of glucagon-like peptide-1 receptor mRNA, protein and binding in the male nonhuman primate (Macaca mulatta) brain. Endocrinology 156, 255–267. 10.1210/en.2014-1675 25380238 PMC4272390

[B79] Heppner K. M. Marks S. Holland J. Ottaway N. Smiley D. Dimarchi R. (2015b). Contribution of brown adipose tissue activity to the control of energy balance by GLP-1 receptor signalling in mice. Diabetologia 58, 2124–2132. 10.1007/s00125-015-3651-3 26049402 PMC4529364

[B80] Hervas I. Queiroz C. M. Adell A. Artigas F. (2000). Role of uptake inhibition and autoreceptor activation in the control of 5-HT release in the frontal cortex and dorsal hippocampus of the rat. Br. J. Pharmacol. 130, 160–166. 10.1038/sj.bjp.0703297 10781012 PMC1572046

[B81] Hestad K. A. Engedal K. Whist J. E. Aukrust P. Farup P. G. Mollnes T. E. (2016). Patients with depression display cytokine levels in serum and cerebrospinal fluid similar to patients with diffuse neurological symptoms without a defined diagnosis. Neuropsychiatr. Dis. Treat 12, 817–822. 10.2147/NDT.S101925 27110115 PMC4835112

[B82] Hill A. S. Sahay A. Hen R. (2015). Increasing Adult Hippocampal Neurogenesis is Sufficient to Reduce Anxiety and Depression-Like Behaviors. Neuropsychopharmacology 40, 2368–2378. 10.1038/npp.2015.85 25833129 PMC4538351

[B83] Hoeffer C. A. Klann E. (2010). mTOR signaling: at the crossroads of plasticity, memory and disease. Trends Neurosci. 33, 67–75. 10.1016/j.tins.2009.11.003 19963289 PMC2821969

[B84] Holscher C. (2010). Incretin analogues that have been developed to treat type 2 diabetes hold promise as a novel treatment strategy for Alzheimer’s disease. Recent Pat. CNS Drug Discov. 5, 109–117. 10.2174/157488910791213130 20337586

[B85] Holscher C. (2012). Potential role of glucagon-like peptide-1 (GLP-1) in neuroprotection. CNS Drugs 26, 871–882. 10.2165/11635890-000000000-00000 22938097

[B86] Holscher C. (2014). Central effects of GLP-1: new opportunities for treatments of neurodegenerative diseases. J. Endocrinol. 221, T31–T41. 10.1530/JOE-13-0221 23999914

[B87] Holst J. J. (2007). The physiology of glucagon-like peptide 1. Physiol. Rev. 87, 1409–1439. 10.1152/physrev.00034.2006 17928588

[B88] Hunter K. Holscher C. (2012). Drugs developed to treat diabetes, liraglutide and lixisenatide, cross the blood brain barrier and enhance neurogenesis. BMC Neurosci. 13, 33. 10.1186/1471-2202-13-33 22443187 PMC3352246

[B89] Irwin M. R. Miller A. H. (2007). Depressive disorders and immunity: 20 years of progress and discovery. Brain Behav. Immun. 21, 374–383. 10.1016/j.bbi.2007.01.010 17360153

[B90] Isacson R. Nielsen E. Dannaeus K. Bertilsson G. Patrone C. Zachrisson O. (2011). The glucagon-like peptide 1 receptor agonist exendin-4 improves reference memory performance and decreases immobility in the forced swim test. Eur. J. Pharmacol. 650, 249–255. 10.1016/j.ejphar.2010.10.008 20951130

[B91] Iwai T. Sawabe T. Tanimitsu K. Suzuki M. Sasaki-Hamada S. Oka J. (2014). Glucagon-like peptide-1 protects synaptic and learning functions from neuroinflammation in rodents. J. Neurosci. Res. 92, 446–454. 10.1002/jnr.23335 24464856

[B92] Jha S. Rajendran R. Fernandes K. A. Vaidya V. A. (2008). 5-HT2A/2C receptor blockade regulates progenitor cell proliferation in the adult rat hippocampus. Neurosci. Lett. 441, 210–214. 10.1016/j.neulet.2008.06.028 18603367

[B93] Jiang H. Ling Z. Zhang Y. Mao H. Ma Z. Yin Y. (2015). Altered fecal microbiota composition in patients with major depressive disorder. Brain Behav. Immun. 48, 186–194. 10.1016/j.bbi.2015.03.016 25882912

[B94] Jourdi H. Hsu Y. T. Zhou M. Qin Q. Bi X. Baudry M. (2009). Positive AMPA receptor modulation rapidly stimulates BDNF release and increases dendritic mRNA translation. J. Neurosci. 29, 8688–8697. 10.1523/JNEUROSCI.6078-08.2009 19587275 PMC2761758

[B95] Kang H. J. Voleti B. Hajszan T. Rajkowska G. Stockmeier C. A. Licznerski P. (2012). Decreased expression of synapse-related genes and loss of synapses in major depressive disorder. Nat. Med. 18, 1413–1417. 10.1038/nm.2886 22885997 PMC3491115

[B96] Kappe C. Tracy L. M. Patrone C. Iverfeldt K. Sjoholm A. (2012). GLP-1 secretion by microglial cells and decreased CNS expression in obesity. J. Neuroinflammation 9, 276. 10.1186/1742-2094-9-276 23259618 PMC3546916

[B97] Kappe C. Patrone C. Holst J. J. Zhang Q. Sjoholm A. (2013). Metformin protects against lipoapoptosis and enhances GLP-1 secretion from GLP-1-producing cells. J. Gastroenterol. 48, 322–332. 10.1007/s00535-012-0637-5 22850868

[B98] Karpova N. N. Pickenhagen A. Lindholm J. Tiraboschi E. Kulesskaya N. Agustsdottir A. (2011). Fear erasure in mice requires synergy between antidepressant drugs and extinction training. Science 334, 1731–1734. 10.1126/science.1214592 22194582 PMC3929964

[B99] Kastin A. J. Akerstrom V. (2003). Entry of exendin-4 into brain is rapid but may be limited at high doses. Int. J. Obes. Relat. Metab. Disord. 27, 313–318. 10.1038/sj.ijo.0802206 12629557

[B100] Kastin A. J. Akerstrom V. Pan W. (2002). Interactions of glucagon-like peptide-1 (GLP-1) with the blood-brain barrier. J. Mol. Neurosci. 18, 7–14. 10.1385/JMN:18:1-2:07 11931352

[B101] Kessler R. C. Chiu W. T. Demler O. Merikangas K. R. Walters E. E. (2005). Prevalence, severity, and comorbidity of 12-month DSM-IV disorders in the National Comorbidity Survey Replication. Arch. Gen. Psychiatry 62, 617–627. 10.1001/archpsyc.62.6.617 15939839 PMC2847357

[B102] Khandaker G. M. Cousins L. Deakin J. Lennox B. R. Yolken R. Jones P. B. (2015). Inflammation and immunity in schizophrenia: implications for pathophysiology and treatment. Lancet Psychiatry 2, 258–270. 10.1016/S2215-0366(14)00122-9 26359903 PMC4595998

[B103] Klempin F. Beis D. Mosienko V. Kempermann G. Bader M. Alenina N. (2013). Serotonin is required for exercise-induced adult hippocampal neurogenesis. J. Neurosci. 33, 8270–8275. 10.1523/JNEUROSCI.5855-12.2013 23658167 PMC6619640

[B104] Knight R. Callewaert C. Marotz C. Hyde E. R. Debelius J. W. Mcdonald D. (2017). The Microbiome and Human Biology. Annu. Rev. Genomics Hum. Genet. 18, 65–86. 10.1146/annurev-genom-083115-022438 28375652

[B105] Kohler S. Thomas A. J. Barnett N. A. O’brien J. T. (2010). The pattern and course of cognitive impairment in late-life depression. Psychol. Med. 40, 591–602. 10.1017/S0033291709990833 19656429

[B106] Kohler C. A. Freitas T. H. Maes M. De Andrade N. Q. Liu C. S. Fernandes B. S. (2017). Peripheral cytokine and chemokine alterations in depression: a meta-analysis of 82 studies. Acta Psychiatr. Scand. 135, 373–387. 10.1111/acps.12698 28122130

[B107] Kondo M. Nakamura Y. Ishida Y. Shimada S. (2015). The 5-HT3 receptor is essential for exercise-induced hippocampal neurogenesis and antidepressant effects. Mol. Psychiatry 20, 1428–1437. 10.1038/mp.2014.153 25403840

[B108] Kopschina Feltes P. Doorduin J. Klein H. C. Juarez-Orozco L. E. Dierckx R. A. Moriguchi-Jeckel C. M. (2017). Anti-inflammatory treatment for major depressive disorder: implications for patients with an elevated immune profile and non-responders to standard antidepressant therapy. J. Psychopharmacol. 31, 1149–1165. 10.1177/0269881117711708 28653857 PMC5606303

[B109] Korn T. Bettelli E. Oukka M. Kuchroo V. K. (2009). IL-17 and Th17 Cells. Annu. Rev. Immunol. 27, 485–517. 10.1146/annurev.immunol.021908.132710 19132915

[B110] Korol S. V. Jin Z. Babateen O. Birnir B. (2015). GLP-1 and exendin-4 transiently enhance GABAA receptor-mediated synaptic and tonic currents in rat hippocampal CA3 pyramidal neurons. Diabetes 64, 79–89. 10.2337/db14-0668 25114295

[B111] Kraus C. Castren E. Kasper S. Lanzenberger R. (2017). Serotonin and neuroplasticity - Links between molecular, functional and structural pathophysiology in depression. Neurosci. Biobehav. Rev. 77, 317–326. 10.1016/j.neubiorev.2017.03.007 28342763

[B112] Krishnan V. Nestler E. J. (2008). The molecular neurobiology of depression. Nature 455, 894–902. 10.1038/nature07455 18923511 PMC2721780

[B113] Ledford H. (2014). Medical research: if depression were cancer. Nature 515, 182–184. 10.1038/515182a 25391943

[B114] Lee Y. S. Park M. S. Choung J. S. Kim S. S. Oh H. H. Choi C. S. (2012). Glucagon-like peptide-1 inhibits adipose tissue macrophage infiltration and inflammation in an obese mouse model of diabetes. Diabetologia 55, 2456–2468. 10.1007/s00125-012-2592-3 22722451

[B115] Lener M. S. Niciu M. J. Ballard E. D. Park M. Park L. T. Nugent A. C. (2017). Glutamate and Gamma-Aminobutyric Acid Systems in the Pathophysiology of Major Depression and Antidepressant Response to Ketamine. Biol. Psychiatry 81, 886–897. 10.1016/j.biopsych.2016.05.005 27449797 PMC5107161

[B116] Lennox R. Porter D. W. Flatt P. R. Gault V. A. (2013). (Val(8))GLP-1-Glu-PAL: a GLP-1 agonist that improves hippocampal neurogenesis, glucose homeostasis, and beta-cell function in high-fat-fed mice. ChemMedChem 8, 595–602. 10.1002/cmdc.201200409 23138973

[B117] Lennox R. Porter D. W. Flatt P. R. Holscher C. Irwin N. Gault V. A. (2014). Comparison of the independent and combined effects of sub-chronic therapy with metformin and a stable GLP-1 receptor agonist on cognitive function, hippocampal synaptic plasticity and metabolic control in high-fat fed mice. Neuropharmacology 86, 22–30. 10.1016/j.neuropharm.2014.06.026 24998752

[B118] Leonard B. E. (2018). Inflammation and depression: a causal or coincidental link to the pathophysiology? Acta Neuropsychiatr. 30, 1–16. 10.1017/neu.2016.69 28112061

[B119] Li X. Jope R. S. (2010). Is glycogen synthase kinase-3 a central modulator in mood regulation? Neuropsychopharmacology 35, 2143–2154. 10.1038/npp.2010.105 20668436 PMC3055312

[B120] Li Y. Perry T. Kindy M. S. Harvey B. K. Tweedie D. Holloway H. W. (2009). GLP-1 receptor stimulation preserves primary cortical and dopaminergic neurons in cellular and rodent models of stroke and Parkinsonism. Proc. Natl. Acad. Sci. U. S. A. 106, 1285–1290. 10.1073/pnas.0806720106 19164583 PMC2633544

[B121] Li Y. Tweedie D. Mattson M. P. Holloway H. W. Greig N. H. (2010). Enhancing the GLP-1 receptor signaling pathway leads to proliferation and neuroprotection in human neuroblastoma cells. J. Neurochem. 113, 1621–1631. 10.1111/j.1471-4159.2010.06731.x 20374430 PMC2912144

[B122] Lietzau G. Magni G. Kehr J. Yoshitake T. Candeias E. Duarte A. I. (2020). Dipeptidyl peptidase-4 inhibitors and sulfonylureas prevent the progressive impairment of the nigrostriatal dopaminergic system induced by diabetes during aging. Neurobiol. Aging 89, 12–23. 10.1016/j.neurobiolaging.2020.01.004 32143981

[B123] Liu H. Dear A. E. Knudsen L. B. Simpson R. W. (2009). A long-acting glucagon-like peptide-1 analogue attenuates induction of plasminogen activator inhibitor type-1 and vascular adhesion molecules. J. Endocrinol. 201, 59–66. 10.1677/JOE-08-0468 19136619

[B124] Llewellyn-Smith I. J. Gnanamanickam G. J. Reimann F. Gribble F. M. Trapp S. (2013). Preproglucagon (PPG) neurons innervate neurochemically identified autonomic neurons in the mouse brainstem. Neuroscience 229, 130–143. 10.1016/j.neuroscience.2012.09.071 23069752 PMC4298013

[B125] Luciani P. Deledda C. Benvenuti S. Cellai I. Squecco R. Monici M. (2010). Differentiating effects of the glucagon-like peptide-1 analogue exendin-4 in a human neuronal cell model. Cell Mol. Life Sci. 67, 3711–3723. 10.1007/s00018-010-0398-3 20496097 PMC11115565

[B126] MacQueen G. Frodl T. (2011). The hippocampus in major depression: evidence for the convergence of the bench and bedside in psychiatric research? Mol. Psychiatry 16, 252–264. 10.1038/mp.2010.80 20661246

[B127] Maes M. Leonard B. E. Myint A. M. Kubera M. Verkerk R. (2011). The new ’5-HT’ hypothesis of depression: cell-mediated immune activation induces indoleamine 2,3-dioxygenase, which leads to lower plasma tryptophan and an increased synthesis of detrimental tryptophan catabolites (TRYCATs), both of which contribute to the onset of depression. Prog. Neuropsychopharmacol. Biol. Psychiatry 35, 702–721. 10.1016/j.pnpbp.2010.12.017 21185346

[B128] Mainardi M. Fusco S. Grassi C. (2015). Modulation of hippocampal neural plasticity by glucose-related signaling. Neural Plast. 2015, 657928. 10.1155/2015/657928 25977822 PMC4419237

[B129] Manigault K. R. Thurston M. M. (2016). Liraglutide: A Glucagon-Like Peptide-1 Agonist for Chronic Weight Management. Consult Pharm. 31, 685–697. 10.4140/TCP.n.2016.685 28074747

[B130] Marchetti I. Koster E. H. Sonuga-Barke E. J. De Raedt R. (2012). The default mode network and recurrent depression: a neurobiological model of cognitive risk factors. Neuropsychol. Rev. 22, 229–251. 10.1007/s11065-012-9199-9 22569771

[B131] Massart R. Mongeau R. Lanfumey L. (2012). Beyond the monoaminergic hypothesis: neuroplasticity and epigenetic changes in a transgenic mouse model of depression. Philos. Trans. R. Soc. Lond. B. Biol. Sci. 367, 2485–2494. 10.1098/rstb.2012.0212 22826347 PMC3405682

[B132] McClean P. L. Holscher C. (2014). Liraglutide can reverse memory impairment, synaptic loss and reduce plaque load in aged APP/PS1 mice, a model of Alzheimer’s disease. Neuropharmacology 76 (Pt A), 57–67. 10.1016/j.neuropharm.2013.08.005 23973293

[B133] McClean P. L. Gault V. A. Harriott P. Holscher C. (2010). Glucagon-like peptide-1 analogues enhance synaptic plasticity in the brain: a link between diabetes and Alzheimer’s disease. Eur. J. Pharmacol. 630, 158–162. 10.1016/j.ejphar.2009.12.023 20035739

[B134] McClean P. L. Parthsarathy V. Faivre E. Holscher C. (2011). The diabetes drug liraglutide prevents degenerative processes in a mouse model of Alzheimer’s disease. J. Neurosci. 31, 6587–6594. 10.1523/JNEUROSCI.0529-11.2011 21525299 PMC6622662

[B135] McEwen B. S. Eiland L. Hunter R. G. Miller M. M. (2012). Stress and anxiety: structural plasticity and epigenetic regulation as a consequence of stress. Neuropharmacology 62, 3–12. 10.1016/j.neuropharm.2011.07.014 21807003 PMC3196296

[B136] McGovern S. F. Hunter K. Holscher C. (2012). Effects of the glucagon-like polypeptide-1 analogue (Val8)GLP-1 on learning, progenitor cell proliferation and neurogenesis in the C57B/16 mouse brain. Brain Res. 1473, 204–213. 10.1016/j.brainres.2012.07.029 22867941

[B137] McKie S. Del-Ben C. Elliott R. Williams S. Del Vai N. Anderson I. (2005). Neuronal effects of acute citalopram detected by pharmacoMRI. Psychopharmacol. (Berl) 180, 680–686. 10.1007/s00213-005-2270-y 15889241

[B138] McQuade R. Sharp T. (1997). Functional mapping of dorsal and median raphe 5-hydroxytryptamine pathways in forebrain of the rat using microdialysis. J. Neurochem. 69, 791–796. 10.1046/j.1471-4159.1997.69020791.x 9231740

[B139] Mendez-David I. Hen R. Gardier A. M. David D. J. (2013). Adult hippocampal neurogenesis: an actor in the antidepressant-like action. Ann. Pharm. Fr. 71, 143–149. 10.1016/j.pharma.2013.02.006 23622692

[B140] Merchenthaler I. Lane M. Shughrue P. (1999). Distribution of pre-pro-glucagon and glucagon-like peptide-1 receptor messenger RNAs in the rat central nervous system. J. Comp. Neurol. 403, 261–280. 10.1002/(SICI)1096-9861(19990111)403:2<261::AID-CNE8>3.0.CO;2-5 9886047

[B141] Miller R. J. Rostene W. Apartis E. Banisadr G. Biber K. Milligan E. D. (2008). Chemokine action in the nervous system. J. Neurosci. 28, 11792–11795. 10.1523/JNEUROSCI.3588-08.2008 19005041 PMC2746239

[B142] Mohammadi M. Nasehi M. Zarrindast M. R. (2015). Modulation of the effects of the cannabinoid agonist, ACPA, on spatial and non-spatial novelty detection in mice by dopamine D1 receptor drugs infused into the basolateral amygdala. Behav. Brain Res. 280, 36–44. 10.1016/j.bbr.2014.11.003 25476564

[B143] Monroe S. M. Harkness K. L. (2005). Life stress, the “kindling” hypothesis, and the recurrence of depression: considerations from a life stress perspective. Psychol. Rev. 112, 417–445. 10.1037/0033-295X.112.2.417 15783292

[B144] Mora P. F. Johnson E. L. (2017). Cardiovascular Outcome Trials of the Incretin-Based Therapies: What Do We Know So Far? Endocr. Pract. 23, 89–99. 10.4158/EP161481.RA 27819769

[B145] Morrison J. H. Baxter M. G. (2012). The ageing cortical synapse: hallmarks and implications for cognitive decline. Nat. Rev. Neurosci. 13, 240–250. 10.1038/nrn3200 22395804 PMC3592200

[B146] Moylan S. Maes M. Wray N. R. Berk M. (2013). The neuroprogressive nature of major depressive disorder: pathways to disease evolution and resistance, and therapeutic implications. Mol. Psychiatry 18, 595–606. 10.1038/mp.2012.33 22525486

[B147] Murrough J. W. Abdallah C. G. Mathew S. J. (2017). Targeting glutamate signalling in depression: progress and prospects. Nat. Rev. Drug Discov. 16, 472–486. 10.1038/nrd.2017.16 28303025

[B148] Najjar S. Pearlman D. M. Devinsky O. Najjar A. Zagzag D. (2013). Neurovascular unit dysfunction with blood-brain barrier hyperpermeability contributes to major depressive disorder: a review of clinical and experimental evidence. J. Neuroinflammation 10, 142. 10.1186/1742-2094-10-142 24289502 PMC4220803

[B149] Neufeld K. A. Foster J. A. (2009). Effects of gut microbiota on the brain: implications for psychiatry. J. Psychiatry Neurosci. 34, 230–231. 19448854 PMC2674977

[B150] Nunes S. O. Reiche E. M. Morimoto H. K. Matsuo T. Itano E. N. Xavier E. C. (2002). Immune and hormonal activity in adults suffering from depression. Braz. J. Med. Biol. Res. 35, 581–587. 10.1590/S0100-879X2002000500011 12011944

[B151] Onoviran O. F. Li D. Toombs Smith S. Raji M. A. (2019). Effects of glucagon-like peptide 1 receptor agonists on comorbidities in older patients with diabetes mellitus. Ther. Adv. Chronic Dis. 10, 2040622319862691. 10.1177/2040622319862691 31321014 PMC6628533

[B152] O’Mahony S. M. Clarke G. Borre Y. E. Dinan T. G. Cryan J. F. (2015). Serotonin, tryptophan metabolism and the brain-gut-microbiome axis. Behav. Brain Res. 277, 32–48. 10.1016/j.bbr.2014.07.027 25078296

[B153] Parker H. E. Reimann F. Gribble F. M. (2010). Molecular mechanisms underlying nutrient-stimulated incretin secretion. Expert Rev. Mol. Med. 12, e1. 10.1017/S146239940900132X 20047700

[B154] Parthsarathy V. Holscher C. (2013a). Chronic treatment with the GLP1 analogue liraglutide increases cell proliferation and differentiation into neurons in an AD mouse model. PLoS One 8, e58784. 10.1371/journal.pone.0058784 23536825 PMC3594148

[B155] Parthsarathy V. Holscher C. (2013b). The type 2 diabetes drug liraglutide reduces chronic inflammation induced by irradiation in the mouse brain. Eur. J. Pharmacol. 700, 42–50. 10.1016/j.ejphar.2012.12.012 23276669

[B156] Paudel Y. N. Shaikh M. F. Shah S. Kumari Y. Othman I. (2018). Role of inflammation in epilepsy and neurobehavioral comorbidities: Implication for therapy. Eur. J. Pharmacol. 837, 145–155. 10.1016/j.ejphar.2018.08.020 30125565

[B157] Perlman G. Simmons A. N. Wu J. Hahn K. S. Tapert S. F. Max J. E. (2012). Amygdala response and functional connectivity during emotion regulation: a study of 14 depressed adolescents. J. Affect. Disord. 139, 75–84. 10.1016/j.jad.2012.01.044 22401827 PMC3340443

[B158] Petrik D. Jiang Y. Birnbaum S. G. Powell C. M. Kim M. S. Hsieh J. (2012). Functional and mechanistic exploration of an adult neurogenesis-promoting small molecule. FASEB J. 26, 3148–3162. 10.1096/fj.11-201426 22542682 PMC3405259

[B159] Piletz J. E. Halaris A. Iqbal O. Hoppensteadt D. Fareed J. Zhu H. (2009). Pro-inflammatory biomakers in depression: treatment with venlafaxine. World J. Biol. Psychiatry 10, 313–323. 10.3109/15622970802573246 19921973

[B160] Porter W. D. Flatt P. R. Holscher C. Gault V. A. (2013). Liraglutide improves hippocampal synaptic plasticity associated with increased expression of Mash1 in ob/ob mice. Int. J. Obes. (Lond.) 37, 678–684. 10.1038/ijo.2012.91 22665137

[B161] Price J. L. Drevets W. C. (2010). Neurocircuitry of mood disorders. Neuropsychopharmacology 35, 192–216. 10.1038/npp.2009.104 19693001 PMC3055427

[B162] Pytka K. Dziubina A. Mlyniec K. Dziedziczak A. Zmudzka E. Furgala A. (2016). The role of glutamatergic, GABA-ergic, and cholinergic receptors in depression and antidepressant-like effect. Pharmacol. Rep. 68, 443–450. 10.1016/j.pharep.2015.10.006 26922551

[B163] Rajkowska G. Miguel-Hidalgo J. J. Wei J. Dilley G. Pittman S. D. Meltzer H. Y. (1999). Morphometric evidence for neuronal and glial prefrontal cell pathology in major depression. Biol. Psychiatry 45, 1085–1098. 10.1016/S0006-3223(99)00041-4 10331101

[B164] Rajkowska G. O’dwyer G. Teleki Z. Stockmeier C. A. Miguel-Hidalgo J. J. (2007). GABAergic neurons immunoreactive for calcium binding proteins are reduced in the prefrontal cortex in major depression. Neuropsychopharmacology 32, 471–482. 10.1038/sj.npp.1301234 17063153 PMC2771699

[B165] Rao M. Gershon M. D. (2016). The bowel and beyond: the enteric nervous system in neurological disorders. Nat. Rev. Gastroenterol. Hepatol. 13, 517–528. 10.1038/nrgastro.2016.107 27435372 PMC5005185

[B166] Rebosio C. Balbi M. Passalacqua M. Ricciarelli R. Fedele E. (2018). Presynaptic GLP-1 receptors enhance the depolarization-evoked release of glutamate and GABA in the mouse cortex and hippocampus. Biofactors 44, 148–157. 10.1002/biof.1406 29265673

[B167] Reed J. Bain S. Kanamarlapudi V. (2020). Recent advances in understanding the role of glucagon-like peptide 1. F1000Res 9, 239. 10.12688/f1000research.20602.1 PMC7137394 32269764

[B168] Richard J. E. Anderberg R. H. Goteson A. Gribble F. M. Reimann F. Skibicka K. P. (2015). Activation of the GLP-1 receptors in the nucleus of the solitary tract reduces food reward behavior and targets the mesolimbic system. PLoS One 10, e0119034. 10.1371/journal.pone.0119034 25793511 PMC4368564

[B169] Robinson A. Lubitz I. Atrakchi-Baranes D. Licht-Murava A. Katsel P. Leroith D. (2019). Combination of Insulin with a GLP1 Agonist Is Associated with Better Memory and Normal Expression of Insulin Receptor Pathway Genes in a Mouse Model of Alzheimer’s Disease. J. Mol. Neurosci. 67, 504–510. 10.1007/s12031-019-1257-9 30635783 PMC6549496

[B170] Rush A. J. Trivedi M. H. Wisniewski S. R. Nierenberg A. A. Stewart J. W. Warden D. (2006). Acute and longer-term outcomes in depressed outpatients requiring one or several treatment steps: a STAR*D report. Am. J. Psychiatry 163, 1905–1917. 10.1176/ajp.2006.163.11.1905 17074942

[B171] Sahay A. Hen R. (2007). Adult hippocampal neurogenesis in depression. Nat. Neurosci. 10, 1110–1115. 10.1038/nn1969 17726477

[B172] Salcedo I. Tweedie D. Li Y. Greig N. H. (2012). Neuroprotective and neurotrophic actions of glucagon-like peptide-1: an emerging opportunity to treat neurodegenerative and cerebrovascular disorders. Br. J. Pharmacol. 166, 1586–1599. 10.1111/j.1476-5381.2012.01971.x 22519295 PMC3419902

[B173] Sanacora G. Zarate C. A. Krystal J. H. Manji H. K. (2008). Targeting the glutamatergic system to develop novel, improved therapeutics for mood disorders. Nat. Rev. Drug Discov. 7, 426–437. 10.1038/nrd2462 18425072 PMC2715836

[B174] Sango K. Utsunomiya K. (2015). Efficacy of glucagon-like peptide-1 mimetics for neural regeneration. Neural Regener. Res. 10, 1723–1724. 10.4103/1673-5374.169611 PMC4705767 26807090

[B175] Schmidt-Hieber C. Jonas P. Bischofberger J. (2004). Enhanced synaptic plasticity in newly generated granule cells of the adult hippocampus. Nature 429, 184–187. 10.1038/nature02553 15107864

[B176] Scott L. V. Clarke G. Dinan T. G. (2013). The brain-gut axis: a target for treating stress-related disorders. Mod. Trends Pharmacopsychiatry 28, 90–99. 10.1159/000343971 25224893

[B177] Serafini G. (2012). Neuroplasticity and major depression, the role of modern antidepressant drugs. World J. Psychiatry 2, 49–57. 10.5498/wjp.v2.i3.49 24175168 PMC3782176

[B178] Setoyama D. Kato T. A. Hashimoto R. Kunugi H. Hattori K. Hayakawa K. (2016). Plasma Metabolites Predict Severity of Depression and Suicidal Ideation in Psychiatric Patients-A Multicenter Pilot Analysis. PLoS One 11, e0165267. 10.1371/journal.pone.0165267 27984586 PMC5161310

[B179] Sharma A. N. Ligade S. S. Sharma J. N. Shukla P. Elased K. M. Lucot J. B. (2015). GLP-1 receptor agonist liraglutide reverses long-term atypical antipsychotic treatment associated behavioral depression and metabolic abnormalities in rats. Metab. Brain Dis. 30, 519–527. 10.1007/s11011-014-9591-7 25023888

[B180] Sheline Y. I. Sanghavi M. Mintun M. A. Gado M. H. (1999). Depression duration but not age predicts hippocampal volume loss in medically healthy women with recurrent major depression. J. Neurosci. 19, 5034–5043. 10.1523/JNEUROSCI.19-12-05034.1999 10366636 PMC6782668

[B181] Slusarczyk J. Trojan E. Chwastek J. Glombik K. Basta-Kaim A. (2016). A Potential Contribution of Chemokine Network Dysfunction to the Depressive Disorders. Curr. Neuropharmacol. 14, 705–720. 10.2174/1570159X14666160219131357 26893168 PMC5050392

[B182] Slyepchenko A. Maes M. Kohler C. A. Anderson G. Quevedo J. Alves G. S. (2016). T helper 17 cells may drive neuroprogression in major depressive disorder: Proposal of an integrative model. Neurosci. Biobehav. Rev. 64, 83–100. 10.1016/j.neubiorev.2016.02.002 26898639

[B183] Smith K. J. Au B. Ollis L. Schmitz N. (2018). The association between C-reactive protein, Interleukin-6 and depression among older adults in the community: A systematic review and meta-analysis. Exp. Gerontol. 102, 109–132. 10.1016/j.exger.2017.12.005 29237576

[B184] Smitha J. S. Roopa R. Sagar B. K. Kutty B. M. Andrade C. (2014). Images in electroconvulsive therapy: ECS dose-dependently increases cell proliferation in the subgranular region of the rat hippocampus. J. ECT 30, 193–194. 10.1097/YCT.0000000000000076 24901429

[B185] Solmaz V. Cinar B. P. Yigitturk G. Cavusoglu T. Taskiran D. Erbas O. (2015). Exenatide reduces TNF-alpha expression and improves hippocampal neuron numbers and memory in streptozotocin treated rats. Eur. J. Pharmacol. 765, 482–487. 10.1016/j.ejphar.2015.09.024 26386291

[B186] Son H. Banasr M. Choi M. Chae S. Y. Licznerski P. Lee B. (2012). Neuritin produces antidepressant actions and blocks the neuronal and behavioral deficits caused by chronic stress. Proc. Natl. Acad. Sci. U. S. A. 109, 11378–11383. 10.1073/pnas.1201191109 22733766 PMC3396528

[B187] Song C. Wang H. (2011). Cytokines mediated inflammation and decreased neurogenesis in animal models of depression. Prog. Neuropsychopharmacol. Biol. Psychiatry 35, 760–768. 10.1016/j.pnpbp.2010.06.020 20600462

[B188] Sorensen G. Reddy I. A. Weikop P. Graham D. L. Stanwood G. D. Wortwein G. (2015). The glucagon-like peptide 1 (GLP-1) receptor agonist exendin-4 reduces cocaine self-administration in mice. Physiol. Behav. 149, 262–268. 10.1016/j.physbeh.2015.06.013 26072178 PMC4668599

[B189] Steinberg E. E. Keiflin R. Boivin J. R. Witten I. B. Deisseroth K. Janak P. H. (2013). A causal link between prediction errors, dopamine neurons and learning. Nat. Neurosci. 16, 966–973. 10.1038/nn.3413 23708143 PMC3705924

[B190] Sutcigil L. Oktenli C. Musabak U. Bozkurt A. Cansever A. Uzun O. (2007). Pro- and anti-inflammatory cytokine balance in major depression: effect of sertraline therapy. Clin. Dev. Immunol. 2007, 76396. 10.1155/2007/76396 18317531 PMC2248234

[B191] Taliaz D. Stall N. Dar D. E. Zangen A. (2010). Knockdown of brain-derived neurotrophic factor in specific brain sites precipitates behaviors associated with depression and reduces neurogenesis. Mol. Psychiatry 15, 80–92. 10.1038/mp.2009.67 19621014 PMC2834321

[B192] Tanti A. Belzung C. (2013). Hippocampal neurogenesis: a biomarker for depression or antidepressant effects? Methodological considerations and perspectives for future research. Cell Tissue Res. 354, 203–219. 10.1007/s00441-013-1612-z 23595256

[B193] Tian L. Gao J. Hao J. Zhang Y. Yi H. O’brien T. D. (2010). Reversal of new-onset diabetes through modulating inflammation and stimulating beta-cell replication in nonobese diabetic mice by a dipeptidyl peptidase IV inhibitor. Endocrinology 151, 3049–3060. 10.1210/en.2010-0068 20444936

[B194] Toni N. Teng E. M. Bushong E. A. Aimone J. B. Zhao C. Consiglio A. (2007). Synapse formation on neurons born in the adult hippocampus. Nat. Neurosci. 10, 727–734. 10.1038/nn1908 17486101

[B195] Tran P. B. Banisadr G. Ren D. Chenn A. Miller R. J. (2007). Chemokine receptor expression by neural progenitor cells in neurogenic regions of mouse brain. J. Comp. Neurol. 500, 1007–1033. 10.1002/cne.21229 17183554 PMC2758702

[B196] Tsai J. Rosenheck R. A. (2016). US Veterans’ Use Of VA Mental Health Services And Disability Compensation Increased From 2001 To 2010. Health Aff. (Millwood) 35, 966–973. 10.1377/hlthaff.2015.1555 27269011

[B197] Turton M. D. O’shea D. Gunn I. Beak S. A. Edwards C. M. Meeran K. (1996). A role for glucagon-like peptide-1 in the central regulation of feeding. Nature 379, 69–72. 10.1038/379069a0 8538742

[B198] Tye K. M. Mirzabekov J. J. Warden M. R. Ferenczi E. A. Tsai H. C. Finkelstein J. (2013). Dopamine neurons modulate neural encoding and expression of depression-related behaviour. Nature 493, 537–541. 10.1038/nature11740 23235822 PMC4160519

[B199] Velmurugan K. Balamurugan A. N. Loganathan G. Ahmad A. Hering B. J. Pugazhenthi S. (2012). Antiapoptotic actions of exendin-4 against hypoxia and cytokines are augmented by CREB. Endocrinology 153, 1116–1128. 10.1210/en.2011-1895 22253425

[B200] Ventorp F. Bay-Richter C. Nagendra A. S. Janelidze S. Matsson V. S. Lipton J. (2017). Exendin-4 Treatment Improves LPS-Induced Depressive-Like Behavior Without Affecting Pro-Inflammatory Cytokines. J. Parkinsons. Dis. 7, 263–273. 10.3233/JPD-171068 28387682 PMC5438473

[B201] Vertes R. P. (1991). A PHA-L analysis of ascending projections of the dorsal raphe nucleus in the rat. J. Comp. Neurol. 313, 643–668. 10.1002/cne.903130409 1783685

[B202] Wang J. W. David D. J. Monckton J. E. Battaglia F. Hen R. (2008). Chronic fluoxetine stimulates maturation and synaptic plasticity of adult-born hippocampal granule cells. J. Neurosci. 28, 1374–1384. 10.1523/JNEUROSCI.3632-07.2008 18256257 PMC6671574

[B203] Wang X. F. Liu J. J. Xia J. Liu J. Mirabella V. Pang Z. P. (2015). Endogenous Glucagon-like Peptide-1 Suppresses High-Fat Food Intake by Reducing Synaptic Drive onto Mesolimbic Dopamine Neurons. Cell Rep. 12, 726–733. 10.1016/j.celrep.2015.06.062 26212334 PMC4860285

[B204] Wang Y. H. Liou K. T. Tsai K. C. Liu H. K. Yang L. M. Chern C. M. (2018). GSK-3 inhibition through GLP-1R allosteric activation mediates the neurogenesis promoting effect of P7C3 after cerebral ischemic/reperfusional injury in mice. Toxicol. Appl. Pharmacol. 357, 88–105. 10.1016/j.taap.2018.08.023 30189238

[B205] Weber C. (2016). Neurogastroenterology: Improving glucose tolerance via the gut-brain axis. Nat. Rev. Gastroenterol. Hepatol. 13, 4. 10.1038/nrgastro.2015.204 26627551

[B206] Weina H. Yuhu N. Christian H. Birong L. Feiyu S. Le W. (2018). Liraglutide attenuates the depressive- and anxiety-like behaviour in the corticosterone induced depression model via improving hippocampal neural plasticity. Brain Res. 1694, 55–62. 10.1016/j.brainres.2018.04.031 29705602

[B207] Wilkinson M. B. Dias C. Magida J. Mazei-Robison M. Lobo M. Kennedy P. (2011). A novel role of the WNT-dishevelled-GSK3beta signaling cascade in the mouse nucleus accumbens in a social defeat model of depression. J. Neurosci. 31, 9084–9092. 10.1523/JNEUROSCI.0039-11.2011 21697359 PMC3133937

[B208] Williams L. M. (2016). Precision psychiatry: a neural circuit taxonomy for depression and anxiety. Lancet Psychiatry 3, 472–480. 10.1016/S2215-0366(15)00579-9 27150382 PMC4922884

[B209] Woelfer M. Kasties V. Kahlfuss S. Walter M. (2019). The Role of Depressive Subtypes within the Neuroinflammation Hypothesis of Major Depressive Disorder. Neuroscience 403, 93–110. 10.1016/j.neuroscience.2018.03.034 29604382

[B210] Yu H. Wang D. D. Wang Y. Liu T. Lee F. S. Chen Z. Y. (2012). Variant brain-derived neurotrophic factor Val66Met polymorphism alters vulnerability to stress and response to antidepressants. J. Neurosci. 32, 4092–4101. 10.1523/JNEUROSCI.5048-11.2012 22442074 PMC3319323

[B211] Yuen E. Y. Wei J. Liu W. Zhong P. Li X. Yan Z. (2012). Repeated stress causes cognitive impairment by suppressing glutamate receptor expression and function in prefrontal cortex. Neuron 73, 962–977. 10.1016/j.neuron.2011.12.033 22405206 PMC3302010

[B212] Zeng L. L. Shen H. Liu L. Wang L. Li B. Fang P. (2012). Identifying major depression using whole-brain functional connectivity: a multivariate pattern analysis. Brain 135, 1498–1507. 10.1093/brain/aws059 22418737

[B213] Zhang R. L. Zhang Z. G. Chopp M. (2008). Ischemic stroke and neurogenesis in the subventricular zone. Neuropharmacology 55, 345–352. 10.1016/j.neuropharm.2008.05.027 18632119 PMC2562038

[B214] Zhang K. Zhu Y. Zhu Y. Wu S. Liu H. Zhang W. (2016). Molecular, Functional, and Structural Imaging of Major Depressive Disorder. Neurosci. Bull. 32, 273–285. 10.1007/s12264-016-0030-0 27142698 PMC5563774

[B215] Zhao J. Bao A. M. Qi X. R. Kamphuis W. Luchetti S. Lou J. S. (2012). Gene expression of GABA and glutamate pathway markers in the prefrontal cortex of non-suicidal elderly depressed patients. J. Affect. Disord. 138, 494–502. 10.1016/j.jad.2012.01.013 22357337

[B216] Zheng P. Zeng B. Zhou C. Liu M. Fang Z. Xu X. (2016). Gut microbiome remodeling induces depressive-like behaviors through a pathway mediated by the host’s metabolism. Mol. Psychiatry 21, 786–796. 10.1038/mp.2016.44 27067014

